# Interaction between Parkin and *α*-Synuclein in *PARK2*-Mediated Parkinson’s Disease

**DOI:** 10.3390/cells10020283

**Published:** 2021-01-31

**Authors:** Daniel Aghaie Madsen, Sissel Ida Schmidt, Morten Blaabjerg, Morten Meyer

**Affiliations:** 1 Department of Neurobiology Research, Institute of Molecular Medicine, University of Southern Denmark, 5000 Odense, Denmark; damadsen@health.sdu.dk (D.A.M.); sischmidt@health.sdu.dk (S.I.S.); Morten.Blaabjerg1@rsyd.dk (M.B.); 2 Department of Neurology, Odense University Hospital, 5000 Odense, Denmark; 3 Department of Clinical Research, University of Southern Denmark, 5000 Odense, Denmark; 4 BRIDGE—Brain Research Inter-Disciplinary Guided Excellence, Department of Clinical Research, University of Southern Denmark, 5000 Odense, Denmark

**Keywords:** familial Parkinson’s disease, *PARK2*, parkin, *α*-synuclein, Lewy bodies

## Abstract

Parkin and *α*-synuclein are two key proteins involved in the pathophysiology of Parkinson’s disease (PD). Neurotoxic alterations of *α*-synuclein that lead to the formation of toxic oligomers and fibrils contribute to PD through synaptic dysfunction, mitochondrial impairment, defective endoplasmic reticulum and Golgi function, and nuclear dysfunction. In half of the cases, the recessively inherited early-onset PD is caused by loss of function mutations in the *PARK2* gene that encodes the E3-ubiquitin ligase, parkin. Parkin is involved in the clearance of misfolded and aggregated proteins by the ubiquitin-proteasome system and regulates mitophagy and mitochondrial biogenesis. *PARK2*-related PD is generally thought not to be associated with Lewy body formation although it is a neuropathological hallmark of PD. In this review article, we provide an overview of post-mortem neuropathological examinations of *PARK2* patients and present the current knowledge of a functional interaction between parkin and *α*-synuclein in the regulation of protein aggregates including Lewy bodies. Furthermore, we describe prevailing hypotheses about the formation of intracellular micro-aggregates (synuclein inclusions) that might be more likely than Lewy bodies to occur in *PARK2*-related PD. This information may inform future studies aiming to unveil primary signaling processes involved in PD and related neurodegenerative disorders.

## 1. Introduction

Parkinson’s disease (PD) is the second most common neurodegenerative disorder and the most prevalent neurodegenerative movement disorder. It is characterized by the progressive loss of neuromelanin-containing dopaminergic neurons in substantia nigra pars compacta (SNpc) [1]. 

PD primarily manifests as a movement disorder with cardinal motor symptoms such as bradykinesia, resting tremor, and rigidity [2]. However, it has become apparent that non-motor symptoms, which include constipation, REM-sleep behavior disorder, and hyposmia, may precede the motor symptoms by several years. Other non-motor symptoms may also occur, and at late disease stages, postural instability and dementia are seen [3]. The motor symptoms appear when approximately 50–60% of the dopaminergic neurons have degenerated, thereby lowering the amount of dopamine in the striatum by 70-80%, which causes dysregulation of basal ganglia activity [4]. 

In industrialized countries, PD affects at least 0.3% of the general population, but this increases with age such that the incidence is 1% in people older than 60 years and over 3% in people older than 80 years [5,6]. While 5–10% of PD patients have a monogenic variant with Mendelian inheritance, the remaining 90–95% is sporadic with unknown etiology [7]. Today, 23 loci and 19 disease-causing genes have been associated with PD, reflecting the heterogeneity in phenotype, age at onset, and inheritance [8]. A mutation in the *PARK2* gene is the most common cause of autosomal recessive PD (ARPD), which accounts for 50% of early-onset parkinsonism [9] and is the main focus of this review article. 

A histological hallmark of PD is the presence of intraneuronal eosinophilic proteinaceous inclusions termed Lewy bodies, which were first described by the German-born American neurologist Friedrich Heinrich Lewy in 1912 [10]. A study in 1997 by Spillantini et al. revealed that α-synuclein was the primary component of Lewy bodies [11]. Since then, proteome studies have shown that Lewy bodies consist of more than 300 proteins, of which approximately 90 have been confirmed by immunohistochemistry in post-mortem studies [12]. However, the process by which Lewy body pathology occurs and their role in the neurodegenerative process in PD remain to be determined. Lewy body pathology is generally thought to be absent in *PARK2*-related PD [13], but it is uncertain whether Lewy bodies are present in *PARK2*-related PD patients. In this review, we summarize the current data describing Lewy body pathology in *PARK2*-related PD, with the main focus on a potential interaction between α-synuclein and parkin in the formation of Lewy bodies or micro-aggregates (synuclein inclusions). 

## 2. *α*-Synuclein

**Figure 1:**
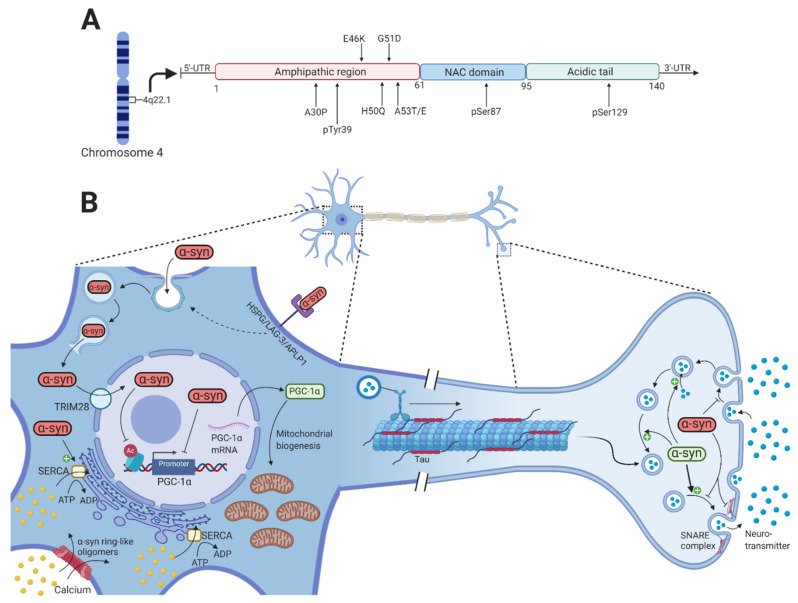
α-synuclein function and pathology. (**A**) Shows a schematic representation of α-synuclein on transcript level with color-coded functional domains and mutations of α-synuclein mentioned in this article. (**B**) Under physiological conditions, α-synuclein (green) is involved in the compartmentalization, storage, and recycling of neurotransmitters. It also contributes to the exocytotic process by promoting SNARE-complex assembly, thereby enabling the fusion of intracellular presynaptic vesicles with the presynaptic membrane. Neurotoxic alterations of α-synuclein (red) by missense mutations in the *SNCA* gene, gene duplication or triplication, and various PTMs increase the formation of toxic oligomers and fibrils that disrupt intracellular processes. The internalization of toxic α-synuclein is through dynamin-mediated endocytosis and cell surface protein-mediated uptake through HSPG, LAG-3, and APLP1. Inside the endosome, α-synuclein is able to rupture the membrane to allow direct entry to the cytosol. α-synuclein oligomers can permeabilize the plasma membrane by creating pore-like structures, thus increasing the cytosolic calcium concentration. Activation of the SERCA pump by these oligomers will later contribute further to the increased cytosolic calcium. Through TRIM28, toxic α-synuclein accumulates inside the nucleus where it binds and inhibits the PGC-1α promoter and reduces histone H3 acetylation, potentially affecting numerous cellular processes. This results in reduced PGC-1α mRNA and protein, which reduces mitochondrial biogenesis. Created with BioRender.com.

In humans, *α*-synuclein is a member of a three-protein family: *α*-synuclein, β-synuclein, and γ-synuclein [14]. It is a small, soluble 140 amino acid protein located in the presynaptic terminal and is highly expressed in the brain and specifically in some of the PD-affected regions such as the hippocampus, olfactory bulb, SNpc, dorsal motor nucleus of the vagus, and the lateral and medial mammillary nucleus [15]. However, α-synuclein is also present outside the central nervous system, particularly in the submandibular gland, enteric nervous system, sympathetic ganglia, cardiac and pelvic plexuses, adrenal medulla, and skin [16,17,18,19,20]. It contains three domains, i.e., an amphipathic N-terminal region (1–60), a non-amyloid component (NAC) (61–95), and a C-terminal region (96–140) [21]. The N-terminal region contains a highly conserved area with 11 amino acid repeats that enable α-synuclein to bind membranes through the formation of an amphipathic α-helix. All the known mutations that lead to pathologically dysfunctional α-synuclein are located in this domain, which emphasizes its importance in α-synuclein pathology [22]. The NAC domain contains a stretch of 12 amino acids that are amyloidogenic and is responsible for the polymerization and aggregation of α-synuclein [23]. The C-terminal region contains many charged amino acids and is the domain that contains most of the posttranslational modification (PTM) sites (Figure 1A) [24].

Numerous normal physiological functions of α-synuclein are known, but there are likely to be many others not yet identified. Recent studies have revealed that α-synuclein contributes to the normal functioning of transmitter compartmentalization, storage, and recycling (Figure 1B) [25]. At the presynaptic terminal, α-synuclein is associated with the reserve pool of synaptic vesicles [26], binds membranes through electrostatic interactions with anionic lipids by lysine residues of the N-terminal domain of α-synuclein [27], and induces membrane curvature by an extended helical structure [28]. Burré et al. showed that α-synuclein promotes the assembly of soluble N-ethylmaleimide-sensitive factor attachment protein receptor (SNARE) complex that involves binding of the N-terminal domain to phospholipids and the C-terminal domain to synaptobrevin-2/vesicle-associated membrane protein 2 (VAMP-2), which directly promotes the fusion of the intracellular presynaptic vesicles with the presynaptic membrane [29]. 

In its native conformation, α-synuclein has been observed to exist as both monomers [30] and tetramers, mediated by its KTKEGV repeats [31]. The PD-associated A53T and A30P mutations in α-synuclein shift the native tetrameric conformation towards the monomeric conformation [32], suggesting that the destabilization of the α-helical tetramers and the increased number of unfolded monomers facilitate the aggregation-mediated pathology of α-synuclein in PD. However, neurotoxic alterations in α-synuclein that lead to the formation of oligomers and fibrils are mediated by missense mutations (A53T, A53E, A30P, E46K, H50Q, and G51D) in the *SNCA* gene [33,34,35,36,37,38,39], gene dosage changes by whole-locus duplications or triplication [40,41], and PTMs of α-synuclein that include phosphorylation of serine 87 (pSer87) and serine 129 (pSer129) [42], oxidation, and nitration (Figure 1A) [43].

### 2.1. α-Synuclein Propagation and Seeding

Three discoveries were particularly crucial in the understanding of α-synuclein-mediated PD pathogenesis and disease spread in the nervous system. These were the finding of a PD-associated mutation in the gene encoding α-synuclein [33], the discovery that α-synuclein is a primary component of Lewy bodies [11], and the investigation of Lewy body pathology in numerous brain autopsies by Braak et al. in 2003 [44]. The latter study showed that as PD advances, Lewy body pathology affects progressively more regions of the nervous system [44]. Two long-term transplantation studies published in 2008 led to the hypothesis that misfolded and aggregated α-synuclein can be propagated to interconnected neurons, resulting in the recruitment and misfolding of endogenous α-synuclein like a prion protein [45,46]. Patients in these studies were either transplanted bilaterally with solid pieces of human ventral mesencephalon [46] in the post-commissural putamen or transplanted with fetal mesencephalic dopaminergic neurons in the putamen [45]. In both studies, however, neuropathological examination showed the presence of α-synuclein-positive Lewy bodies in the long-surviving grafted neurons as well as abundant disease-related pSer129 α-synuclein [45,46]. Since then, extensive efforts have been made to discover the underlying mechanisms of the α-synuclein-mediated spreading to interconnected neurons. 

First and foremost, different exocytotic pathways including non-classical exocytosis [47], exosomal release [48,49,50], and direct penetration from the cell membrane [51] have been shown to mediate the release of toxic α-synuclein oligomers and fibrils to the extracellular space. Increased stress [52] as well as mitochondrial and proteasomal dysfunction [47] were associated with an increased release of toxic α-synuclein oligomers and fibrils [47,52]. Different mechanisms for the internalization of α-synuclein oligomers and fibrils from the extracellular space have been intensively studied in recent years and include endocytosis, micropinocytosis, and cell surface protein-mediated uptake [53]. The endocytosis of α-synuclein fibrils has been shown to be facilitated by dynamin [54]. Inside the endosome, α-synuclein is able to rupture the endosomal membrane, thereby evading lysosomal degradation and creating direct entry into the cytosol [55]. Furthermore, heparan sulfate proteoglycan (HSPG), lymphocyte activation gene-3 (LAG-3), neurexin 1b, and amyloid-beta precursor-like protein 1 (APLP1) mediates the uptake of α-synuclein fibrils by micropinocytosis [56,57]. Therefore, multiple cell surface receptors might mediate the internalization of α. -synuclein fibrils. 

These findings highlight the different molecular mechanisms that lead to release of α-synuclein oligomers and fibrils to the extracellular space and their uptake by interconnected neurons. This was verified in studies demonstrating that the introduction of exogenous α-synuclein fibrils results in the seeding and recruitment of soluble endogenous α-synuclein leading to the formation of Lewy bodies in human cell lines [58] and mouse hippocampal neurons [59]. 

Having described the ability of toxic α-synuclein oligomers and fibrils to propagate and seed in interconnected neurons, we will now look at how normal cellular homeostasis is disrupted.

### 2.2. α-Synuclein-Mediated Toxicity

Numerous research articles have described multiple dysfunctional pathways associated with α-synuclein toxicity in PD pathogenesis. These include dysfunctional synaptic-vesicle trafficking, impaired mitochondrial function, defective endoplasmic reticulum, and Golgi function, defective autophagy-lysosomal pathway, and nuclear dysfunction [60]. 

Regarding the dysfunctional synaptic-vesicle trafficking, it has been shown that large α-synuclein oligomers preferentially bind to synaptobrevin-2/VAMP2, thereby preventing SNARE complex formation and the fusion of dopamine-containing presynaptic vesicles with the presynaptic membrane (Figure 1B) [61]. Furthermore, overexpression of α-synuclein in a range corresponding to *SNCA* gene multiplication resulted in inhibition of neurotransmitter release and a reduced presynaptic vesicle recycling pool size [62]. Although α-synuclein seems to be mainly located in the presynaptic terminal, it has also been shown in the nucleus [63]. PD-associated mutations in α-synuclein as well as PTMs (such as pSer129 α-synuclein) and oxidative stress increase its nuclear localization compared to wild-type α-synuclein [64,65,66]. The accumulation of α-synuclein in the nucleus is mediated by the nuclear protein TRIM28 [67]. Once inside the nucleus, α-synuclein appears to bind the promoter of the mitochondrial transcription activator peroxisome proliferator-activated receptor gamma-coactivator 1α (PGC1α) both in vitro, in vivo, and in brain tissue of PD patients, which leads to reduced activity of the PGC1α promoter and reduced levels of PGC1α mRNA and protein [68,69]. Furthermore, the nuclear localization of α-synuclein reduces acetylation of histone H3 as part of the neurotoxicity in the nucleus [66]. The nuclear localization of α-synuclein might thus result in mitochondrial dysfunction, which is one of the main hallmarks of PD, and impair other pathways whose dysfunction might contribute to PD pathogenesis. 

Addition of prefibrillar α-synuclein oligomers have been reported to result in calcium-induced mitochondrial swelling, mitochondrial depolarization, and cytochrome c release, thereby impairing mitochondrial homeostasis and potentially initiating apoptosis [70,71]. Aggregated but not monomeric α-synuclein is able to bind and activate the SERCA pump, leading to an initial reduction in the cytosolic calcium concentration that is followed by a later increase [72]. Other studies have reported a marked increase in the cytosolic calcium concentration upon increased α-synuclein expression, leading to a toxic activation of a calcium-calmodulin (CaM)-calcineurin cascade [73,74]. The increased cytosolic calcium concentration can be explained by studies demonstrating the ability of α-synuclein oligomers to permeabilize lipid bilayers by creating pore-like structures that cause structural alterations in both the intracellular and plasma membrane. This will result in calcium flux from the extracellular space and intracellular stores to the cytosol, thereby activating the CaM-calcineurin cascade leading to toxic effects [75,76]. 

Regarding the defective autophagy-lysosomal pathway, the accumulation of α-synuclein reduces lysosomal degradation capacity in induced pluripotent stem cell (iPSC)-derived neurons and human midbrain dopamine models through reduced activity of glucocerebrosidase and β-galactosidase; the cause has been suggested to be dysfunctional trafficking of lysosomal enzymes from the endoplasmic reticulum [77,78,79]. The precise sequence of intracellular mechanisms of α-synuclein-mediated neurotoxicity that lead to neuronal death in PD remains inconclusive, however.

## 3. Parkin

**Figure 2:**
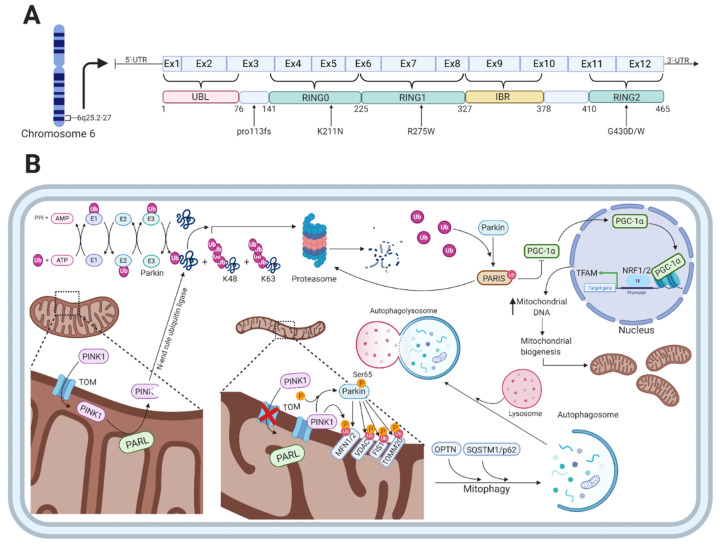
The function of parkin and *PARK2*-related mutations. (**A**) Shows a schematic representation of parkin on transcript level, color-coded functional domains, and *PARK2*-related mutations mentioned in this article. (**B**) Shows the various cellular functions of parkin, which is involved in the ubiquitin-proteasome system as an E3-ubiquitin ligase, regulation of mitophagy, and mitochondrial biogenesis. In healthy mitochondria, PINK1 is transported through the TOM complex to the inner mitochondrial membrane, which is followed by cleavage by PARL. The PINK1 protein fragment is released to the cytosol where it gets ubiquitylated by an N-end rule ubiquitin ligase for proteasomal degradation. With damaged mitochondria, PINK1 accumulates on the outer mitochondrial membrane bound to the TOM complex. This leads to PINK1-mediated phosphorylation of parkin at serine 65 in its UBL-domain that increases the activity of parkin. The ubiquitylation of proteins on the OMM by parkin includes MFN1/2, VDAC, FIS1, and TOMM20, which is sensed by autophagic cargo receptors OPTN and SQSTM1/p62. Additionally, the parkin-mediated ubiquitylation and degradation of PARIS (that normally represses PGC-1α) results in activation of the transcription factors NRF1/2 that will initiate the transcription of mitochondrial probiogenesis factors such as TFAM, thereby increasing mitochondrial biogenesis. Created with BioRender.com

The *PARK2* gene encodes the 52kDa protein parkin, which consists of 12 exons and 465 amino acids [80,81]. A mutation in the *PARK2* gene causes an autosomal recessive form of PD and is the most frequent cause of early-onset PD (EOPD) found in several different families with distinct ethnicities [82]. Parkin functions as an E3-ubiquitin ligase and has a broad range of neuroprotective functions including the maintenance of mitochondrial metabolism [83] and the ubiquitin-proteasome system, where parkin plays an essential role in the ubiquitin-mediated degradation of misfolded or damaged proteins and in removal of dysfunctional mitochondria via mitophagy [84]. The protein is widely expressed throughout the brain, and abundant expression of parkin mRNA has been observed in other tissues such as the heart and skeletal muscles [81]. Parkin is a RING-in-between-RING (RBR)-type E3 ubiquitin ligase that catalyzes the mono- and poly-ubiquitylation of several structurally and functionally distinct proteins, including itself [85,86]. Parkin consists of an N-terminal ubiquitin-like (UBL) domain that is followed by four cysteine-rich regions, each of which binds two Zn^2+^ atoms [87]. Three of those regions are the really interesting new gene (RING) domains designated as RING0-2, the last two of which are separated by a 51-residue in-between-RING (IBR) domain in the C-terminal part (Figure 2A) [88].

More than 100 PD-associated mutations in the 12 exons of the *PARK2* gene have been identified including missense mutations, large chromosomal deletions and duplications, truncation mutations, and promoter mutations [82,89]. Besides mutations that impair the function of the parkin protein, PTMs such as S-nitrosylation, covalent binding of dopamine, phosphorylation by the stress-activated kinase c-Abl, and oxidative stress have been shown to impair the activity of parkin in sporadic PD [90,91,92,93,94,95,96]. Interestingly, carriers of PD-associated *PARK2* mutations have very similar clinical phenotypes to patients with sporadic PD [97]. In fact, it is not possible based on clinical symptoms alone to distinguish PD patients with parkin mutations from those with sporadic PD [98]. However, more distinctive clinical phenotypes associated with *PARK2* mutations include earlier age of onset, frequent dystonia, and hyperreflexia, and slower disease progression despite the early onset (see Table 1 for a more comprehensive overview) [97]. Additionally, *PARK2*-associated PD patients have a good response towards Levodopa treatment but are prone to develop Levodopa-mediated dyskinesia [99,100]. Pathologically, *PARK2*-associated PD patients show a significant reduction of neurons in the SNpc and only a moderate decrease of neurons in locus coeruleus [101].

### Parkin Function

Parkin functions as an E3-ubiquitin ligase that is engaged in monoubiquitylation [104] and multiple monoubiquitylation [105] as well as K48-linked and K63-linked polyubiquitylation [106]. The classical K48-linked polyubiquitylation targets substrates for the proteasomal degradation [107], whereas the K63-linked polyubiquitylation plays a proteasomal-independent role in the regulation of protein trafficking for lysosomal degradation and targeting whole organelles for autophagic degradation [108]. The proteasomal-mediated degradation of proteins involves the sequential action of three enzymes, i.e., ubiquitin-activating (E1), ubiquitin-conjugating (E2), and ubiquitin-ligase (E3), which cooperate in adding ubiquitin molecules to proteins destined for degradation (Figure 2B) [109]. Through a sequential and repetitive action of these enzymes, ubiquitin molecules are attached to substrate proteins via a covalent isopeptide between the glycine at residue 76 (G76) in the C-terminal part of ubiquitin and lysine at residue 48 (K48) at the N-terminal part of the substrate protein targeting them for proteasomal degradation [110,111]. 

Besides the function of parkin in the ubiquitin proteasomal system, several studies have shown that parkin specifically translocates from the cytosol to dysfunctional mitochondria upon an impaired electrochemical membrane potential leading to mitochondrial depolarization [112,113,114]. Mitophagy, the autophagy-mediated degradation of mitochondria, is in part mediated by the PD-associated proteins parkin and PINK1 in a ubiquitin-dependent mechanism (Figure 2B) [113,115]. In the parkin-dependent mitophagy process, PINK1 functions as the mitochondrial damage sensor, parkin as a signal amplifier, and the ubiquitin chains as the signal effector [116]. When healthy mitochondria are present, PINK1 is transported through the translocase of the outer membrane (TOM) complex to the inner mitochondrial membrane (IMM), mediated by its N-terminal mitochondrial targeting sequence [117]. This is followed by its subsequent cleavage by presenilin-associated rhomboid-like protein (PARL), a protease in the IMM, which leads to the production of a 52kDa PINK1 protein fragment [118,119]. The protein fragment is released to the cytosol where it is rapidly ubiquitylated for proteasomal degradation by an N-end rule ubiquitin ligase [118]. Thus, the intracellular levels of PINK1 are low on healthy mitochondria. 

When mitochondria are damaged, they become depolarized, leading to inhibition of PINK1 translocation and processing in the IMM [120]. This results in the accumulation of unprocessed PINK1 bound to the TOM complex on the outer mitochondrial membrane (OMM) and the subsequent phosphorylation of serine 65 in the UBL-domain of parkin, which increases the ubiquitin chain assembly and hence parkin activity [121,122]. The parkin-mediated ubiquitylation of proteins located on the OMM includes mitofusin 1/2 (MFN1/2), voltage-dependent anion-selective channel (VDAC) proteins, mitochondrial fission 1 protein (FIS1), and mitochondrial import receptor subunit TOM20 homologue (TOMM20) [107]. The formation of these chains of ubiquitin molecules on OMM proteins is sensed by autophagic cargo receptors such as optineurin (OPTN) and sequestosome 1 (SQSTM1/p62) that will initiate the degradation of damaged mitochondria [123]. 

In contrast to parkin’s role in the clearance of dysfunctional and damaged mitochondria, it also mediates mitochondrial biogenesis (Figure 2B). Under physiological conditions, parkin indirectly regulates the expression of the mitochondrial transcriptional coactivator PGC-1α [124]. This is done through the ability of parkin to degrade the parkin interacting substrate (PARIS), which normally inhibits the activity of PGC-1α [124]. Stabilization and activation of PGC-1α through the parkin-mediated degradation of PARIS leads to activation of the transcription factors nuclear respiratory factor 1 and 2 (NRF1/2), which will switch on mitochondrial probiogenesis factors such as mitochondrial transcription factor A (TFAM) [125,126]. Loss of parkin function, which is the case in *PARK2*-related PD, results in the accumulation of PARIS and, therefore, to sustained repression of PGC-1α activity [127]. This will eventually lead to the inhibition of mitochondrial biogenesis. 

Proteomic, structural, and functional analyses were applied in a recent study using human isogenic iPSC-derived neurons with and without a *PARK2* knockout (KO) [128]. This study identified dysregulation of several proteins in the *PARK2* KO neurons that are involved in oxidative stress, mitochondrial respiration and morphology, cell cycle control, and cell viability [128]. Furthermore, the structural and functional analyses showed accumulation of enlarged and elongated mitochondria, which is consistent with the function of parkin in mitochondrial quality control [128]. These functions of parkin highlight its pivotal role in the production and degradation of mitochondria as well as its essential function in the ubiquitin-proteasome system. 

Why has *PARK2*-related PD with its classical PD pathology and phenotypes not been associated with Lewy body formation? In the following section, we will examine the literature on post-mortem neuropathological investigations of *PARK2* patients to provide an overview of neuropathological findings.

## 4. Lewy Body Pathology in PARK2-Related PD

Homozygous and compound heterozygous mutations in the *PARK2* gene are an established cause of heritable EOPD [97]. In contrast, heterozygous missense mutations may predispose to late-onset PD, which resembles sporadic PD, but it remains elusive how and whether missense mutations in the parkin protein contribute to the pathophysiology of PD [129,130]. Ambiguous results have been obtained when investigating Lewy body pathology in post-mortem brain sections of *PARK2* patients. Therefore, we performed a comprehensive literature analysis to examine current knowledge, the results of which are presented in Table 2. 

Seventeen studies published from 1994 to 2018 have investigated post-mortem brain sections from *PARK2* patients with a focus on the neuropathology and Lewy body formation. Of the seventeen reported cases, fifteen were homozygous or compound heterozygous, and the remaining two cases were heterozygous. The presence of Lewy bodies was reported in eight cases (of which seven showed *PARK2* patients with typical Lewy bodies [131,132,133,134,135,136,137] and one showed basophilic Lewy body-like inclusion bodies in the neuropils of the pedunculopontine nucleus in the mesencephalic reticular formation [138]), while ten cases had no signs of Lewy body deposition [101,135,139,140,141,142,143,144,145,146,147]. Besides the presence of intraneuronal Lewy bodies, nine of the seventeen post-mortem studies revealed the neuropathological presence of tau pathology [101,133,134,135,137,142,143,146,147]. 

The initial work connecting EOPD pathology to the *PARK2* gene came from studies in Japan [148]. The clinical features of EOPD such as early age of onset, parkinsonism with diurnal fluctuation, good response towards Levodopa, dystonia, hyperreflexia, absence of dementia, and a relatively benign disease course were first described by Yamamura et al. in 1973 [149]. A screening program to identify the gene responsible for EOPD was initiated in 1993 by the Department of Neurology at Juntendo University. In 1997, this screening led to the identification of the gene locus 6q25.2–27 as being responsible for EOPD [150]. Finally, in 1998, Kitada et al. discovered the novel gene encoding parkin that was linked to locus 6q25.2–27, and EOPD was later designated as *PARK2* [81].

The first post-mortem brain section analysis with a focus on *PARK2*-mediated neuropathology and Lewy body formation was reported by Yamamura et al. in 1993 [140,141]. The neuropathological analysis showed a highly depigmented SNpc and intense gliosis, but no Lewy bodies were seen. Furthermore, a slightly decreased number of pigmented neurons were observed in the ventral tegmental areas and locus coeruleus. However, no pathology was observed in substantia nigra pars reticulata (SNpr), striatum, pallidum, thalamus, nucleus basalis of Meynert, or raphe nuclei [140,141]. A year later, Takahashi et al. showed similar neuropathological changes in EOPD patients [139]. Although the connection between the gene locus and parkin was first discovered in 1998, the studies and neuropathological findings from Yamamura et al. and Takahashi et al. are included in this article as patients from both cases have been confirmed in later studies to be linked to the 6q25.2–27 locus [151]. 

Three post-mortem brain sections of *PARK2* patients carrying a homozygous exon 4 deletion have been reported in the literature [101,142,145]. These patients developed PD before the age of 40 years, and neuropathological investigations showed moderate to severe loss of neurons in SNpc and locus coeruleus without Lewy body pathology. Neurofibrillary tangles in the cerebral cortex and brainstem nuclei as well as tau pathology in hippocampus, frontal, temporal, and parietal cortices, were observed in one of the studies [101]. 

Pathological examination of a Tunisian homozygous patient carrying a two-base AG deletion in exon 2 was reported by Gouider-Khouja et al. in 2003 [144]. The patient developed resting tremor at the age of 34 years and died at age 47. The brain autopsy showed a moderate cell loss in SNpc with a corresponding gliosis. Neither Lewy bodies nor tau or ubiquitin-positive inclusions were observed [144]. In addition to the homozygous cases in which Lewy bodies were not detected, Van de Warrenburg et al. presented a compound heterozygous case with an exon 6 K211N missense mutation and exon 3 deletion in 2001 [143]. Despite the early age of onset (18 years), the patient had a long course of disease and died aged 75. The post-mortem examination showed loss of neurons in both SNpc and the spinocerebellar system while tau pathology was reported in caudate nucleus, putamen, subthalamic nucleus, and substantia nigra. However, no Lewy body pathology was observed [143].

In 2001, Farrer et al. were the first to report classical Lewy body pathology with a corresponding loss of neurons in SNpc and locus coeruleus in a compound heterozygous *PARK2* patient with an exon 3 deletion and exon 7 R275W missense mutation [131]. In 2004, Sasaki et al. reported a homozygous *PARK2* patient with an exon 3 deletion [138]. Pathological investigation showed moderate to severe depletion of neurons in SNpc and a mild depletion in locus coeruleus. Lewy body-like basophilic inclusions were observed in the neuropils of the pedunculopontine nucleus in the mesencephalic reticular formation [138]. In 2005, Pramstaller et al. described a case from a large pedigree where the compound heterozygous patient carried an exon 7 deletion and a deletion of nucleotide T1072 [132]. Neuropathological examination revealed the presence of α-synuclein-positive Lewy bodies as well as the typical neuron depletion in SNpc and locus coeruleus. Ruffmann et al. [133] in 2012 and Miyakawa et al. [134] in 2013 described two unusual *PARK2*-associated cases with late onset. Ruffmann et al. described a heterozygous patient with an exon 7 R275W missense mutation and onset at 62 years, whereas Miyakawa et al. described a homozygous deletion of exon 2 to 4 with onset at 61 years. Ruffmann et al. found severe depletion of neurons from SNpc and locus coeruleus but numerous α-synuclein immunoreactive Lewy bodies whose distribution throughout the brain was compatible with Braak stage VI [133]. Miyakawa et al. also found severe depletion of neurons and the presence of Lewy bodies in SNpc and locus coeruleus, but the distribution of Lewy bodies in the brain was compatible with Braak stage IV [134]. These studies described the presence of pre-tangles in subiculum, transentorhinal, and entorhinal cortex and tau pathology in the entorhinal cortex [133,134].

Two subsequent studies were reported by Doherty et al. [135] and Selikhova et al. [136] in 2013. Doherty et al. [135] presented five different compound heterozygous cases where all showed moderate to severe loss of neurons in SNpc and mild to moderate loss of neurons in locus coeruleus. Lewy body pathology was only observed in cases 3 and 5. Deposition of hyperphosphorylated tau was also observed in case 3 and severe amyloidβ (Aβ) diffuse deposits were seen in case 1 and 4, whereas mild deposits were seen in case 5 [135]. The genotype in cases 3 and 5 with the Lewy bodies was R275W/G430W and R275W/exon 6 deletion. Selikhova et al. reported a case with a compound heterozygous R275W mutation and exon 6 deletion. Neuropathological examination revealed moderate loss of neurons in SNpc with corresponding sparse Lewy body deposition in transentorhinal cortex and cingulate gyrus [136]. The latest reported case with Lewy body pathology was by Sharp et al. in 2014 [137]. This case carried a heterozygous exon 3-4 deletion, developed hand tremor at the age of 44 years, memory loss at age 66, and died at age 76. The post-mortem examination revealed severe loss of neurons in SNpc and the presence of classical Lewy bodies in hippocampus, putamen, and ambient gyrus compatible with Braak stage VI. There was mild occurrence of neuronal tangles (stage 1/6), but no Aβ was observed [137]. 

In 2015, Cornejo-Olivas and colleagues reported a case in a Peruvian family with EOPD that showed a novel splice site mutation in intron 5 (IVS5-1G>A) and an exon 7 deletion [146]. The autopsy revealed severe neuronal loss in SNpc and loss of tyrosine hydroxylase-positive fibers in the striatum, but neither Lewy body pathology nor α-synuclein-positive inclusions were observed [146]. Neurofibrillary tangles compatible with Braak stage II were seen, however [146]. The most recent reported case of post-mortem neuropathological changes in *PARK2* patients was described by Johansen et al. in 2018 [147]. Their case carried a homozygous deletion of exon 3-4 that resulted in the development of PD at the age of 20 years. The patient died at age 79, and the brain autopsy showed severe neuronal loss and gliosis in SNpc with scanty tau pathology but no Lewy body pathology [147]. 

Interpretation of these neuropathological examinations of *PARK2* patients show highly divergent findings. What could be the reason for these conflicting results, and what hypothesis could support the observed findings? In the following section, we will try to unravel the explanations for the neuropathological findings.

### 4.1. What Could Be the Reason for the Ambiguous Post-Mortem Results?

A neuropathological diagnosis of PD requires two distinct pathological criteria. The first is loss of neuromelanin-containing dopaminergic neurons in the SNpc with a corresponding intact striatum, which is the projection target from the SNpc [152]. Secondly, Lewy body pathology should be present [153,154]. Despite advanced methods for clinical diagnosis and the current understanding of the pathophysiological intracellular mechanism leading to PD, there is still a clinical-pathological discordance [155]. It has also become clear that even with the same parkin genotype in patients from the same family, the correlation between the clinical phenotype and the molecular pathology is inconsistent [156]. 

Examination of the post-mortem brain studies presented in Table 2 indicates that although *PARK2*-mediated PD is not thought to be associated with Lewy body pathology, 8 of 17 cases (corresponding to 47%) reported classical Lewy body pathology [131,132,133,134,135,136,137] despite one of the cases reporting Lewy body-like inclusions [138]. What could be the reason for this disparity in the observation of Lewy body pathology between the post-mortem studies? Several hypotheses have been stated in the literature related to Lewy bodies in *PARK2*-related PD. One possibility is that the Lewy bodies observed in post-mortem studies represent incidental Lewy body pathology as they are frequently found in healthy older individuals [157,158,159]. Secondly, patients with late disease onset might have a dysfunctional protein clearance system to remove dysfunctional and accumulated proteins [160]. A third possibility is that some parkin mutations might result in residual parkin activity, leading to an increased probability of Lewy body formation [135,160]. 

It is noteworthy that the 10 Lewy body-negative cases presented in Table 2 had a lower mean age of disease onset (25.5 years) than the eight cases with *PARK2*-related α-synuclein and Lewy body pathology (mean 46.3 years). Thus, *PARK2* patients with Lewy body pathology were on average 21 years older at disease onset. Furthermore, the comprehensive literature review did not reveal any *PARK2* cases of juvenile-onset with post-mortem observations of Lewy body pathology. The hypothesis proposed by Doherty and Hardy may be correct, therefore—that *PARK2* patients with younger age of onset may have a more effective protein clearing system or a different mechanism for dealing with abnormal accumulated proteins [160]. This would result in post-mortem neuropathological findings of neuronal loss and gliosis but no Lewy bodies, which is consistent with the literature.

A particularly intriguing finding is that four of the eight cases with Lewy body pathology were heterozygous or compound heterozygous patients with the R275W missense mutation [131,133,135,136]. Furthermore, the presence of the mutation seems to be associated with later disease onset [135]. The R275W mutation is located within the RING finger 1 domain of the parkin protein (see Figure 2A), which normally mediates protein-protein interactions with ubiquitination-associated E2-conjugating proteins UbcH7 and UbcH8 [161]. The R275W mutation has been shown to preserve the E3 ligase activity of parkin and thereby the ability to ubiquitylate substrate proteins [162], and to produce cytoplasmic and nuclear aggresomes [163]. 

The reported cases of Lewy body pathology in *PARK2*-associated PD might thus be due to residual E3 ligase activity of parkin and the ability of the protein to ubiquitylate substrate proteins that contribute to Lewy body formation. Exon deletions in parkin that result in a total loss of its RING finger 1 domain function lead to its inability to ubiquitylate substrate proteins and to form Lewy bodies [129]. 

It could be speculated whether the partial loss of function by the R275W mutation explain a later age of disease onset and the observation of Lewy bodies, whereas exon deletions causing total loss of function explain an earlier age of disease onset and the lack of Lewy bodies, both of which are consistent with the literature.

### 4.2. Are Lewy Bodies Neuroprotective or Not?

As the clinical-pathological expression of PD symptoms does not seem to be dependent on Lewy body formation [164], it has been proposed that the presence of Lewy bodies might be an epiphenomenon rather than a primary event in the *PARK2*-related PD pathogenesis [97]. Several cell culture studies have shown the involvement of parkin in the formation of aggresome-like inclusions through K63-linked ubiquitylation of proteins in Lewy bodies [165,166,167,168]. These findings are consistent with the previously presented hypothesis that specific missense mutations, such as R275W that leads to residual parkin activity, can increase the probability of Lewy body formation whereas exon deletion causing total loss of parkin function leads to lack of Lewy body formation. 

The conversion of α-synuclein monomers to toxic oligomers and fibrils is accelerated by PTMs such as pSer129 [169]. In fact, 90% of the total α-synuclein in Lewy bodies is phosphorylated at serine 129, whereas only 5% of α-synuclein in non-diseased brains contains the similar PTM [43,170,171]. Since normally functioning parkin seems to be required for the development of Lewy body pathology, it has been postulated that the parkin-mediated inclusion formation is a neuroprotective effect to circumvent α-synuclein release to the extracellular space and thus to prevent the spreading of pSer129 α-synuclein fibrils to interconnected neurons [58,59,172,173,174,175]. In line with this hypothesis, three conceptual frameworks of protein aggregation as an underlying mechanism in PD have been described by Alberto J. Espay and colleagues [176]. First, accumulation of α-synuclein could enhance other pathogenic mechanisms leading to neurodegeneration. Secondly, the protein aggregates could be byproducts caused by several pathogenic mechanisms and that the aggregates themselves neither have a pathogenic or a protective role. Thirdly, the sequestration of toxic soluble protein aggregates into insoluble forms could be a neuroprotective mechanism to circumvent neuronal and synaptic dysfunction, thereby, delaying the neurodegenerative process and allow the neuron to function for decades before becoming too overwhelmed [176]. However, it remains to be determined whether the formation of Lewy bodies is neuroprotective or not.

## 5. The Functional Interaction between Parkin and *α*-Synuclein

### 5.1. Parkin Influences Posttranslational Modifications of α-Synuclein

**Figure 3:**
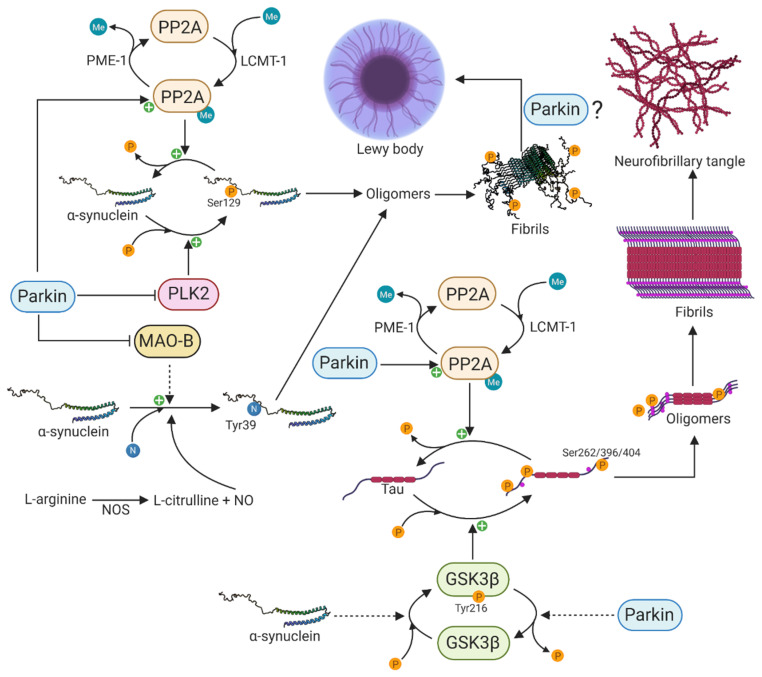
Functional interaction between α-synuclein and parkin in protein aggregation. Parkin can prevent protein aggregation and neurofibrillary tangles through its activation of PP2A. PP2A is able to dephosphorylate both pSer262/396/404 tau and pSer129 α-synuclein, thereby attenuating the formation of toxic oligomers and eventually Lewy bodies and neurofibrillary tangles. Parkin is also able to inhibit the activity of PLK2 that normally phosphorylates α-synuclein on serine 129. Inhibition of MAO-B by parkin results in decreased oxidative stress and decreased formation of Tyr39 nitrated α-synuclein and oligomer formation. Parkin and α-synuclein have opposite functions regarding the activity of the major kinase GSK3β that phosphorylates tau. The phosphorylation of GSK3β at Tyr216 is dependent on α-synuclein, whereas its dephosphorylation is indirectly regulated by parkin. Created with BioRender.com.

α-synuclein undergoes extensive PTMs including phosphorylation, ubiquitination, truncation, and nitration, and many of these have been identified in Lewy bodies, suggesting that these modifications might be necessary and play a primary role in α-synuclein aggregation and neurotoxicity [177]. The disparity in the percentage of pSer129 α-synuclein between brains of PD patients and those of healthy controls suggests a tight regulation under physiological conditions and that the phosphorylation of serine 129 of α-synuclein occurs in conjunction with dopaminergic neuronal cell death in PD [43,170,171]. Additionally, there is growing interest in pSer129 α-synuclein due to its marked accumulation in the brains of PD patients and patients with synucleinopathies [170,171]. The protein phosphatase 2A (PP2A) and polo-like kinase 2 (PLK2) are major regulators of pSer129 α-synuclein. PP2A is a major serine/threonine phosphatase in the brain and is composed of a catalytic C-subunit, a scaffold-like A subunit, and different regulatory B-subunits that confer substrate specificity [178]. Specifically, the B55α-containing isoform of PP2A has been shown to be the major enzyme that dephosphorylates pSer129 α-synuclein [179]. The methylation status of the catalytic C-subunit, which is regulated by leucine carboxyl methyltransferase-1 (LCMT-1)-mediated methylation and protein phosphatase methylesterase-1 (PME-1)-mediated demethylation, specifies the activity of PP2A [180,181]. In 2010, Khandelwal and colleagues investigated the *in vivo* effect of parkin expression on α-synuclein aggregation and pSer129 α-synuclein, using a Lentiviral-mediated gene transfer model [182]. The Lentiviral-mediated expression of α-synuclein resulted in cell death and inflammation as well as increased expression of PLK2 and glycogen synthase kinase 3β (GSK3β), thereby increasing the phosphorylation of α-synuclein and tau, respectively [182]. Interestingly, parkin expression attenuated cell death and inflammation, decreased the levels of PLK2 and GSK3β, and increased the expression of PP2A, leading to decreased levels of pSer129 α-synuclein and phosphorylated tau (Figure 3) [182]. This study clearly shows the essential physiological function of parkin to counteract α-synuclein toxicity and tau hyperphosphorylation. 

In 2016, Park et al. investigated the levels of the PP2A methylating enzyme LCMT-1 and demethylating enzyme PME-1 in post-mortem brains of PD patients [179]. The study demonstrated a significant reduction in the level of LCMT-1 and a significant increase in the level of PME-1 in PD patients and dementia with Lewy bodies (DLB) patients compared to healthy controls, thereby showing decreased activity of PP2A in diseased brains [179]. Furthermore, the methylated to demethylated PP2A ratio was decreased despite no changes in the total amount of PP2A, or the substrate specificity conferring B55α isoform was observed [179]. It remains to be determined, however, what neuroprotective effect parkin might have through potential changes in the levels of LCMT-1 and PME-1. This is essential to our understanding of the molecular mechanisms underlying α-synuclein pathology in *PARK2*-related PD.

Besides undergoing phosphorylation as a potential part of the PD pathogenesis, α-synuclein can also undergo nitration on all four tyrosine residues (Tyr39, Tyr125, Tyr133, and Tyr136)[183]. The neuroinflammation in PD is accompanied by a nitric oxide synthase (NOS)-mediated increased production of nitric oxide (NO). The overexpression of NOS also leads to nitration of α-synuclein and the formation of toxic oligomers in neurons (Figure 3) [184], and nitrated α-synuclein has been observed in Lewy bodies of synucleinopathies including PD [185]. Furthermore, the overexpression of monoamine oxidase B (MAO-B) resulted in a nine-fold increase in 3-nitrotyrosine at Tyr39 of α-synuclein, leading to its oligomerization [186]. Jiang et al. demonstrated in 2006 that parkin suppresses the transcription and expression of MAO-B [187]. Later, in 2012 [188], they used iPSC-derived patient-specific midbrain dopaminergic neurons to investigate the connection between parkin and MAO-B expression. Upon loss of parkin, they observed increased transcription of MAO-B and correspondingly increased oxidative stress, whereas the lentiviral-mediated expression of wild type parkin was able to lower the expression and activity of MAO-B [188]. This clearly shows the essential function of parkin in preventing α-synuclein nitration, oligomerization, and oxidative stress through reduced MAO-B activity.

### 5.2. Parkin Function in α-Synuclein-Mediated Tau Pathology

The accumulation of aggregated α-synuclein in Lewy bodies is the neuropathological hallmark of PD and DLB, whereas the accumulation of aggregated microtubule-associated tau in neurofibrillary tangles is a common feature of Alzheimer’s disease and frontotemporal dementia [189]. Although these two distinct proteins contribute to two different neurodegenerative diseases, there is increasing interest in their potential interaction and mutual aggregation-mediated modulation, which might be an underlying disease-accelerating molecular mechanism in PD [190]. The first experimental evidence leading to the hypothesis linking α-synuclein and tau in a common pathological molecular mechanism was the observation of phosphorylated tau and α-synuclein in neurofibrillary tangles and Lewy bodies in patients with PD and DLB [191,192]. Since then, several lines of evidence have strengthened the hypothesis of a mutual molecular interaction. It has been shown that α-synuclein stimulates the protein kinase A (PKA)-mediated serine 262 (Ser262) phosphorylation of tau that is located in the microtubule-binding region, resulting in microtubule destabilization and neurotoxicity [193,194]. Additionally, a cellular model using 1-methyl-4-phenyl-1,2,3,6-tetrahydropyridine (MPTP) showed that the levels of α-synuclein, rather than the neurotoxic effect of MPTP, were pivotal for the amount of phosphorylated Ser262 [195]. MPTP exerts its neurotoxic effect first by being taken up by astrocytes and converted to the toxic metabolite MPP+ by MAO-B. MPP+ is a substrate for the dopamine transporter leading to the selective degeneration of dopaminergic neurons. Accumulation of MPP+ in dopaminergic neurons result in oxidative stress through inhibition of complex I respiration in mitochondria [196]. GSK3β, in particular, has been shown to be a key protein in the hyperphosphorylation of tau at residue Ser262, Ser396, and Ser404 in an α-synuclein-dependent manner (Figure 3) [197]. This effect seems to be partly due to increased GSK3β kinase activity. Interestingly, Duka et al. [197] revealed that a tyrosine 216 (Tyr216)-dependent phosphorylation of GSK3β was necessary for its activation and that the phosphorylation of GSK3β at Tyr216 and subsequent hyperphosphorylation of tau at Ser262, Ser396, and Ser404 by GSK3β was dependent on α-synuclein [197].

Hyperphosphorylation of tau at Ser262, Ser396, and Ser404 has been shown to be attenuated by the PD-associated parkin protein in several studies [182,198] through decreased phosphorylation of GSK3β at the activation-associated Tyr216 leading to its inhibition [198]. Furthermore, the decrease in tau phosphorylation was dependent on the presence of intracellular α-synuclein [198]. This observation was supported in a study by Khandelwal and colleagues, who also reported that activation of PP2A by parkin resulted in the dephosphorylation of tau [182]. However, it still remains to be determined how parkin affects the activity of PP2A and GSK3β. 

The presented studies suggest a link between α-synuclen, tau, GSK3β, PP2A, PLK2, and parkin as an underlying disease mechanism in *PARK2*-related PD. These studies also demonstrate an essential and neuroprotective function of parkin in α-synuclein and tau pathology. The loss of parkin and its neuroprotective function in *PARK2*-related PD might potentially result in protein misfolding, accumulation, and aggregation. The hypotheses that parkin activity is required for Lewy body formation but prevents α-synuclein misfolding and aggregation may, at first sight, seem contradictory. However, this inconsistency will be accounted for in later sections.

### 5.3. The Regulation of Apoptosis by Parkin and α-Synuclein

One of the neuropathological criteria in the diagnosis of PD is the selective loss of dopaminergic neurons in the SNpc. Having accounted for potential mechanisms in the regulation of protein aggregation in *PARK2*-related PD, we will now describe possible mechanisms leading to cell death upon loss of parkin function. The main mechanism of dopaminergic neuronal loss in PD resembles apoptosis with the identification of apoptotic chromatin changes and DNA fragmentation in post-mortem studies [199,200]. Elevated levels of caspase 1, 3, 8, and 9 upon neuronal loss in the SNpc of PD patients is also seen [201,202,203]. Interestingly, parkin can prevent the activation of caspase 3 in a p53-dependent way, and ChIP experiments have shown that parkin physically interacts with the p53 promoter, leading to lowered p53 mRNA and protein levels [204]. Parkin thus functions as a p53 transcriptional repressor, and parkin mutations resulting in its loss of function increase the expression of p53. Under physiological conditions, α. -synuclein will down-regulate p53, thereby preventing initiation of apoptosis [205]. However, α-synuclein aggregation seen in PD leads to its inability to inhibit p53, which will enhance p53 expression and potentially lead to cell death [205]. Overexpression of α-synuclein leads to attenuation of NFκB activation in a dose-dependent manner, downregulates the expression of the anti-apoptotic factor Bcl-2, and upregulates GSK3β levels [206]. GSK3β contributes to cell death through its regulation of the anti-apoptotic proteins Bcl-2 and Mcl-1 and the pro-apoptotic protein Bax [207]. Activation of GSK3β has been shown to upregulate and phosphorylate Bax at serine 163, which facilitate its mitochondrial localization [208,209]. Furthermore, GSK3β phosphorylates Mcl-1 at serine 159, leading to its ubiquitylation and degradation [210]. Taken together, the α-synuclein-mediated activation of GSK3β leads to an increased Bax-mediated pore formation in the mitochondrial membrane and sequestering Bcl-2, which results in the release of cytochrome c to the cytosol. Once in the cytosol, cytochrome c binds Apaf-1, which activates procaspase 9 that further activates downstream executioner caspases that initiate the apoptotic process [207,211].

As described in this review, parkin is able to decrease the expression and activity of GSK3β [182,198]. Thus, the loss of parkin function in *PARK2*-related PD might not only promote protein aggregation but also contribute to apoptosis-mediated cell death. Furthermore, GSK3β seems to play a pivotal role in the regulation of both protein aggregation and cell death, a mechanism that might be central in PD with a loss of parkin function.

### 5.4. Micro-Aggregates Instead of Lewy Bodies?

The pivotal role of parkin as an E3-ubiquitin ligase that mediates protein degradation through the proteasome or autophagic system might suggest an essential function in the clearance of aggregated and misfolded proteins. In fact, a dysfunctional proteasome system that leads to accumulation and aggregation of intracellular proteins such as Aβ, tau, and α-synuclein could be the mechanism interconnecting neurodegenerative diseases [190]. Several different studies have shown that protection from overexpression of α-synuclein, tau, and Aβ as well as proteasome inhibition can be mediated by parkin [182,198,212]. Furthermore, parkin overexpression can lead to increased activity of proteasomal enzymes [213]. Proteasomal inhibition resulted in the formation of cytoplasmic noncytotoxic inclusions, however, even in cells overexpressing parkin, which could suggest a proteasomal-dependent activity of parkin [214]. These studies demonstrate an essential function of parkin to increase the proteasome system and degrade misfolded and aggregated proteins. Due to the presence of ubiquitylated α-synuclein in Lewy bodies, it was suggested that there might be a direct interaction between parkin and α-synuclein in the formation of Lewy bodies and potentially the degradation of α-synuclein by the proteasome system [215]. However, only O-glycosylated α-synuclein has shown to be a parkin substrate [216]. It is evident that parkin indirectly affects α-synuclein aggregation and accumulation, which might attenuate α-synuclein-mediated toxicity. In the following section, we present a comprehensive literature review of investigations into the effect of parkin overexpression or deletion on α-synuclein toxicity in primary cell culture or animal models (see Table 3). When comparing the hypotheses, (1) Lewy body formation is dependent on parkin activity or residual parkin activity as described for the clinical observations (Table 2) and (2) parkin prevents α-synuclein misfolding and aggregation as described earlier, the two mechanisms may, at first sight, seem contradictory. Interconnecting the hypotheses might be needed to explain both the clinical and cellular observations. Fifteen studies published from 2002 to 2019 have investigated how either parkin depletion or overexpression affects α-synuclein toxicity. Of the fifteen studies, eleven showed a functional interaction between parkin and α-synuclein [182,217,218,219,220,221,222,223,224,225,226], and four studies did not [227,228,229,230]. An *in vitro* study by Petrucelli et al. in 2002 investigated whether parkin overexpression would be neuroprotective in a primary cell culture overexpressing mutated α-synuclein [217]. They showed that the toxicity associated with overexpression of mutated α-synuclein, which could be mimicked by proteasome inhibition, could be mitigated by overexpression of parkin. Interestingly, no neuroprotection was observed when they used R42P mutated parkin (which lacks ubiquitination activity) [217]. A year later, Goldberg and colleagues generated a mouse model with a germline disruption in parkin [227]. These *PARK2*^−/−^ mice exhibited an increased extracellular concentration of dopamine in the striatum due to increased release, but no decrease in dopaminergic neurons in SNpc was observed. Furthermore, quantification of CD-Crel-1, synphilin-1, and α-synuclein did not show increased amounts of these proteins, suggesting that accumulation and aggregation of α-synuclein do not occur in *PARK2*^−/−^ mice [227]. Two other studies by Lorenzetti et al. in 2004 [228] and Ko et al. in 2005 [229] quantified wild-type α-synuclein in *PARK2*^−/−^ mice, consistent with the results from Goldberg and colleagues. Lorenzetti et al. generated a mouse mutant *quaking*^viable^ (*qk*^v^) model and concluded that the parkin gene and the parkin co-regulator gene (*pacrg*) were located in the deleted interval [228], whereas Ko et al. generated a parkin exon 7 null mouse model [229]. Lorenzetti et al. observed no loss of neurons in SNpc, which is consistent with results obtained by Goldberg et al. but inconsistent with neuropathological observations in EOPD-associated *PARK2* patients. No accumulation of α-synuclein was observed in the studies by Lorenzetti et al. [228] and Ko et al. [229]. 

Using *Drosophila melanogaster* as the organism, three of the fifteen studies investigated a possible functional interaction between parkin and α-synuclein [218,219,222]. In 2003, the study by Yang et al. demonstrated that the co-expression of parkin resulted in the attenuation of A53T and A30P mutated α-synuclein toxicity in *Drosophila melanogaster* [218]. In 2004 and 2006, two studies by Haywood et al. showed that wild-type or A30P mutated α-synuclein-induced loss of climbing ability of *Drosophila melanogaster* could be suppressed by the co-expression of parkin [219,222]. 

Three of the fifteen studies investigated the ameliorative effect of parkin on α-synuclein-induced toxicity in a rat model; two studies used a lentiviral gene delivery system [182,220] and one used the recombinant adeno-associated viral (rAAV) vector system [221]. Discrepancies were seen in the results from Lo Bianco et al. in 2004 [220] and Khandelwal et al. in 2010 [182], both of which used a lentiviral gene delivery system. Lo Bianco et al. reported that the lentiviral-mediated overexpression of parkin mitigated A30P-mutated α-synuclein-induced toxicity and also preserved tyrosine hydroxylase positive cells in the SNpc. This observation was supplemented with the detection of an increased amount of pSer129 α-synuclein in the presence of parkin compared to the amount in rats injected only with A30P-mutated α-synuclein [220]. Khandelwal et al. reported that the lentiviral-mediated overexpression of parkin mitigated the toxicity caused by overexpression of wild-type α-synuclein. However, in contrast to Lo Bianco et al. they found a decreased amount of pSer129 α-synuclein as well as attenuation of cell death and inflammation upon overexpression of parkin [182]. Supplementary analysis revealed that the overexpression of parkin increased the activity of PP2A and decreased the levels and activity of PLK2 and GSK3β, respectively [182]. The ameliorative effect of rAAV vector-mediated parkin overexpression on α-synuclein-induced toxicity was verified in the study by Yamada et al. in 2005 [221]. 

In 2006, Von Coelln et al. [230] analyzed transgenic parkin null mice overexpressing human A53T-mutated α-synuclein. Surprisingly, this showed that parkin deficiency did not impact the age-dependent progression of the neurodegenerative phenotype or the ubiquitination, processing, or solubility of α-synuclein in the transgenic mice [230]. Another unexpected relationship between parkin and α-synuclein was reported in 2009 by Fournier et al. [224] in a study using transgenic parkin null mice overexpressing A30P-mutated α-synuclein. They observed delayed motor impairment and a decreased proportion of pSer129-containing neurons upon parkin deficiency in the transgenic mice [224]. Another investigation of the effect of loss of parkin function on α-synuclein-induced toxicity was performed by Van Rompuy et al. in 2015 [225]. They observed no difference in loss of dopaminergic neurons and increased proportion of pSer129 α-synuclein (total α-synuclein was unchanged) in SNpc upon rAAV vector-mediated overexpression of human wild-type α-synuclein [225]. Similar results were obtained by Yasuda et al. in 2007 [223] using rAAV vector-mediated overexpression of human wild-type α-synuclein and parkin in Macaque monkeys. Co-expression of parkin resulted in attenuation of α-synuclein aggregation and decreased amount of pSer129 α-synuclein [223]. The most recent investigation of the functional interaction between parkin and α-synuclein was by Wilkaniec et al. in 2019 [226]. Using rat pheochromocytoma (PC12) cells with parkin overexpression or parkin knock-down, they found that overexpression of parkin attenuated extracellular α-synuclein oligomer-induced toxicity [226]. 

Although the post-mortem neuropathological examinations of *PARK2* patients are ambiguous regarding the presence of Lewy body pathology, the findings of a functional interaction between parkin and α-synuclein seem highly consistent. Having described the functions of parkin in the modulation of α-synuclein accumulation, PTM-mediated aggregation, and attenuation of α-synuclein-mediated toxicity, we hypothesize that α-synuclein aggregation leading to the formation of intracellular micro-aggregates might be an underlying intracellular pathogenic mechanism in *PARK2*-related PD that is more common than the formation of Lewy bodies. Better understanding of this potential protein aggregation mechanism might reveal how PD could be molecularly interconnected to other neurodegenerative diseases.

## 6. Concluding Remarks and Future Directions

Twenty-two years have passed since Kitada et al. discovered in 1998 the causative parkin gene responsible for EOPD that was designated as *PARK2* [81]. Since then, numerous research articles have been published to elucidate the molecular mechanisms underlying *PARK2*-related PD [151]. The pivotal role of parkin as an E3-ubiquitin ligase involves its function in the clearance of misfolded and aggregated proteins by the proteasome system [231], its regulation of mitophagy [115,232] and mitochondrial biogenesis [233] to prevent mitochondrial dysfunction, and its direct and indirect functions to prevent oxidative stress (Figure 2B) [187,234]. The molecular mechanisms underlying *PARK2*-related PD are complex and involve multiple processes that remain to be determined. Research is needed to understand the dysfunctional intracellular signaling processes as this will initiate the development of novel therapeutic treatments. 

Homozygous and compound heterozygous mutations in the *PARK2* gene that cause early-onset heritable ARPD are generally thought not to be associated with Lewy body formation. As presented in Table 2, eight of the seventeen post-mortem neuropathological examinations of *PARK2* patients revealed the presence of Lewy bodies, and the R275W mutation was present in four of these cases. The R275W mutation has been shown to cause residual parkin activity, however, suggesting that the parkin function is essential in the formation of Lewy bodies. We hypothesize that the loss of parkin function in *PARK2*-related PD may result in an ongoing process with the formation of toxic α-synuclein oligomers and fibrils that disrupts numerous intracellular signaling processes. The loss of parkin also abolishes the formation of Lewy bodies, which could potentially be neuroprotective. Micro-aggregates will continue to be formed, however, leading to severe loss of dopaminergic neurons without the formation of Lewy bodies. 

Due to the presence of Lewy bodies in SNpc and locus coeruleus, where extensive neuronal loss is observed in PD, it has been considered that Lewy bodies are related to the neurodegenerative process. Furthermore, the number of Lewy bodies correlates with the severity of neuronal loss [235]. It should be kept in mind, however, that the observation of Lewy bodies in dying neurons does not necessarily mean they are mediators of the lethal effect as they could just be the product of a pathological process [236]. The parkin-mediated formation of Lewy bodies could be a neuroprotective mechanism to prevent the spreading and seeding of toxic pSer129 α-synuclein oligomers and fibrils to interconnected neurons and other brain regions. An indirect functional interaction between parkin and α-synuclein seems to exist in the formation of pSer129 phosphorylated α-synuclein and eventually oligomers, fibrils, and potentially Lewy bodies as their opposite regulation in the formation of hyperphosphorylated tau and eventually the formation of neurofibrillary tangles (Figure 3). The second neuropathological criterion for a diagnosis of PD, which is the presence of Lewy bodies, does not seem to be very specific or of diagnostic value as Lewy bodies have been identified in other neurodegenerative diseases such as Alzheimer’s disease and in brains of healthy older individuals. Based on the presented theory of an indirect functional interaction between parkin and α-synuclein, it seems more reasonable that proteinaceous micro-aggregates or intermediate species in the aggregation process occur, rather than Lewy body formation, in *PARK2*-related PD with a total loss of parkin function. This process is likely to involve the PTM-regulating kinases, PLK-2 and GSK3β, as well as the phosphatase PP2A. The loss of parkin function, which potentially leads to increased activity of PLK-2, GSK3β, and MAOB and decreased activity of PP2A, might create a self-fueling vicious cycle that results in oxidative stress, nitration, and hyperphosphorylation of α-synuclein and tau creating toxic oligomers and fibrils that disrupt cellular homeostasis (Figure 3). How parkin promotes the formation of Lewy bodies in the final step from oligomers and fibrils remains to be determined, however. The fact that parkin is part of the ubiquitin-proteasome system strengthens the hypothesis that a dysfunctional proteasome system resulting in protein aggregation contributes to *PARK2*-related PD [237]. 

Accumulating evidence in recent years suggests that parkin dysfunction might be involved in the pathogenesis of other neurodegenerative diseases such as Alzheimer’s disease [238]. As stated earlier, parkin protects against overexpression of α-synuclein, tau, Aβ, and proteasome inhibition [182,198,212]. Moreover, as presented in Table 2, nine of the seventeen post-mortem neuropathological studies revealed the presence of tau pathology and/or neurofibrillary tangles, clearly suggesting that loss of parkin function precipitates tau pathology [101,133,134,135,137,142,143,146,147]. Indeed, parkin null mice accumulated high levels of tau [239], and overexpression of parkin in APP/PS1 transgenic mice improved hippocampal long-term potentiation and decreased Aβ load and inflammation [240]. 

It is evident that dysfunctional molecular pathways involving parkin may be relevant to other neurodegenerative diseases such as Alzheimer’s disease. Discovering the pathological intracellular signaling processes in *PARK2*-related PD, with a focus on parkin and α-synuclein, might unveil related dysfunctional pathways where impaired protein clearance systems and imbalances in protein PTMs leading to protein misfolding and aggregation might be a common denominator.

## Figures and Tables

**Table 1:** Clinical phenotypes frequently reported in PD cases with PARK2 mutations [97,100,102,103].

Clinical Phenotype	
Motor features	Bradykinesia
	Resting tremor
Non-motor features	Anxiety
	Psychosis
	Panic attack
	Depression
Other clinical features	Normal cognitive function
	Hyperreflexia
	Frequent focal dystonia
	Sleep benefit
	Benign disease course
	Excellent response to low dose Levodopa
	Prone to develop Levodopa-induced dyskinesias

**Table 2:** Summary of post-mortem neuropathological findings from patients with various *PARK2* mutations.

References	PARK2 Genotype	Age of Onset	Age of Death	Neuropathology	Lewy Body Pathology
[139]	No details reported	NA	67	Loss of neurons and gliosis in SNpc and locus coeruleus.	No
[140,141]	Homozygous deletion between exon 3 and 7.	20	52	Loss of neurons in SNpc and gliosis.	No
[101]	Homozygous exon 4 deletion.	24	62	Moderate loss of neurons in SNpc and locus coeruleus. Tau pathology in hippocampus and neurofibrillary tangles in the frontal, temporal, and parietal cortices.	No
[142]	Homozygous exon 4 deletion	32	70	Loss of neurons and gliosis in SNpc and locus coeruleus. Few Neurofibrillary tangles observed.	No
[131]	Exon 7 R275W missense mutation and exon 3 deletion	41	52	Loss of neurons in SNpc and locus coeruleus.	Yes
[143]	Exon 6 K211N missense mutation and exon 3 deletion.	18	75	Loss of neurons in SNpc and the spinocerebellar system. Tau pathology in caudate nucleus, putamen, subthalamic nucleus, and substantia nigra was reported, but neurofibrillary tangles were absent.	No
[144]	Homozygous two-base AG deletion in exon 2	34	47	Loss of neurons in SNpc and SNpr with astrocytic gliosis.	No
[138]	Homozygous exon 3 deletion	33	70	Moderate to severe depletion of neurons in SNpc and gliosis. Only a mild decrease of neurons in locus coeruleus.	LB in the pedunculopontine nucleus.
[132]	Exon 7 deletion + T1072 deletion	49	73	Reactive gliosis and loss of neurons in SNpc and locus coeruleus.	Yes
[145]	Homozygous exon 4 deletion	24	73	Loss of neurons and gliosis in SNpc and locus coeruleus.	No
[133]	Heterozygous R275W mutation in exon 7.	62	80	Severe loss of neurons in SNpc and locus coeruleus. Pre-tangles observed in subiculum, transentorhinal, and entorhinal cortex.	Yes
[134]	Homozygous deletion of exon 2 to 4.	61	72	A marked decrease in neurons of SNpc and locus coeruleus. Tau pathology was observed in the entorhinal cortex.	Yes
[135]	Case 1: R275W + exon 6 deletion	36	86	Moderate to severe loss of neurons in SNpc in all cases and mild to moderate loss of neurons in locus coeruleus. Hyperphosphorylated tau deposition observed in case 3.	Only in case 3 and 5.
	Case 2: R275W + pro113fs	25	62		
	Case 3: R275W + G430W	33	60		
	Case 4: G430D + pro113fs	32	68		
	Case 5: R275W + exon 6 deletion	46	82		
[136]	Exon 7 R275W missense mutation and exon 6 deletion	48	82	Moderate loss of neurons in SNpc.	Yes
[137]	Heterozygous exon 3–4 deletion.	44	76	Severe loss of neurons in SNpc and locus coeruleus. Presence of neurofibrillary tangles.	Yes
[146]	Splice site mutation in intron 5 (IVS5-1G>A) and exon 7 deletion.	16	60	Severe loss of neurons in SNpc. Neurofibrillary tangle pathology was observed.	No
[147]	Homozygous deletion of exon 3–4.	20	79	Severe neuronal loss and gliosis in SNpc. Scanty tau pathology was observed.	No

**Table 3:** Summary of experiments investigating parkin function on α-synuclein-induced toxicity in vivo and in vitro.

References	Method	Organism	Finding	Parkin and α
[217]	Overexpression of parkin with A53T and A30P mutated α-synuclein	Primary cell culture	Increased parkin expression can mitigate mutated α-synuclein induced toxicity.	Yes
[227]	Quantification of wild-type α-synuclein pathology in parkin^−/−^ mice	Mouse model	No loss of nigral dopaminergic neurons and no accumulation of α-synuclein.	No
[218]	Co-expression of parkin with A53T, A30P, or wild-type α-synuclein	*Drosophila melanogaster*	Increased parkin expression can mitigate mutated α-synuclein induced toxicity.	Yes
[219]	Co-expression of parkin with wild-type α-synuclein.	*Drosophila melanogaster*	Increased parkin expression can suppress α-synuclein-induced loss of climbing ability.	Yes
[228]	Quantification of wild-type α-synuclein pathology in parkin^−/−^ mice	Mouse mutant quaking (viable) model	No loss of dopaminergic neurons and no accumulation of α-synuclein.	No
[220]	Overexpression of wild-type rat parkin with human A30P α-synuclein using lentiviral vector delivery.	Rat model	Increased parkin expression can significantly reduce α-synuclein-induced neuropathology.	Yes
[229]	Quantification of wild-type α-synuclein pathology in parkin^−/−^ mice	Mouse model	No accumulation of α-synuclein.	No
[221]	Overexpression of human wild-type α-synuclein and human parkin in the substantia nigra in rats using rAAV vector	Rat model	Increased parkin expression can mitigate α-synuclein induced toxicity.	Yes
[222]	Co-expression of parkin with A30P α-synuclein.	*Drosophila melanogaster*	Increased expression of parkin could counteract the effect of A30P mutated α-synuclein.	Yes
[230]	Overexpression of human A53T α-synuclein and deletion of parkin.	Mouse model	No effect of loss of parkin on neuropathology.	No
[223]	Co-expression of parkin with wild-type α-synuclein.	Macaque monkeys.	Overexpression of parkin was associated with less accumulation of α-synuclein and phosphorylation at S129.	Yes
[224]	A transgenic mouse model expressing human A30P α-synuclein and no parkin.	Mouse model	A lower amount of pSer129 α-synuclein in parkin^−/−^ mice compared with parkin^+/+^ mice.	Yes
[182]	Co-expression of parkin with wild-type α-synuclein.	Sprague Dawley rats	Increased expression of parkin resulted in a decreased expression of pSer87 and pSer129 α-synuclein.	Yes
[225]	Overexpression of human wild-type α-synuclein by rAAV vectors in parkin^−/−^ and wild type mice.	Mouse model	Increased pSer129 α-synuclein but no loss of dopaminergic neurons in parkin knockout mice.	Yes
[226]	Addition of exogenous α-synuclein oligomers in a primary cell culture with a parkin knock-down or overexpression.	Rat pheochromocytoma (PC12) cells	Parkin overexpression protected against the extracellular α-synuclein oligomer-mediated toxicity.	Yes

## References

[1] Balestrino R., Schapira A.H.V. (2020). Parkinson disease. Eur. J. Neurol..

[2] Henderson M.X., Trojanowski J.Q., Lee V.M.Y. (2019). α-Synuclein pathology in Parkinson’s disease and related α-synucleinopathies. Neurosci. Lett..

[3] Poewe W., Seppi K., Tanner C.M., Halliday G.M., Brundin P., Volkmann J., Schrag A.-E., Lang A.E. (2017). Parkinson disease. Nat. Rev. Dis. Primers.

[4] Pickrell A.M., Youle R.J. (2015). The roles of PINK1, parkin, and mitochondrial fidelity in Parkinson’s disease. Neuron.

[5] Lee A., Gilbert R.M. (2016). Epidemiology of Parkinson Disease. Neurol. Clin..

[6] Draoui A., El Hiba O., Aimrane A., El Khiat A., Gamrani H. (2020). Parkinson’s disease: From bench to bedside. Rev. Neurol. (Paris).

[7] Lunati A., Lesage S., Brice A. (2018). The genetic landscape of Parkinson’s disease. Rev. Neurol. (Paris).

[8] Del Rey N.L.-G., Quiroga-Varela A., Garbayo E., Carballo-Carbajal I., Fernández-Santiago R., Monje M.H.G., Trigo-Damas I., Blanco-Prieto M.J., Blesa J. (2018). Advances in Parkinson’s disease: 200 years later. Front. Neuroanat..

[9] Barodia S.K., Creed R.B., Goldberg M.S. (2017). Parkin and PINK1 functions in oxidative stress and neurodegeneration. Brain Res. Bull..

[10] Rodrigues e Silva A.M., Geldsetzer F., Holdorff B., Kielhorn F.W., Balzer-Geldsetzer M., Oertel W.H., Hurtig H., Dodel R. (2010). Who was the man who discovered the “Lewy bodies”?. Mov. Disord..

[11] Spillantini M.G., Schmidt M.L., Lee V.M., Trojanowski J.Q., Jakes R., Goedert M. (1997). Alpha-synuclein in Lewy bodies. Nature.

[12] Shahmoradian S.H., Lewis A.J., Genoud C., Hench J., Moors T.E., Navarro P.P., Castaño-Díez D., Schweighauser G., Graff-Meyer A., Goldie K.N. (2019). Lewy pathology in Parkinson’s disease consists of crowded organelles and lipid membranes. Nat. Neurosci..

[13] Savitt J.M., Dawson V.L., Dawson T.M. (2006). Diagnosis and treatment of Parkinson disease: Molecules to medicine. J. Clin. Investig..

[14] Xu L., Pu J. (2016). Alpha-synuclein in Parkinson’s disease: From pathogenetic dysfunction to potential clinical application. Parkinson’s Dis..

[15] Taguchi K., Watanabe Y., Tsujimura A., Tanaka M. (2019). Expression of α-synuclein is regulated in a neuronal cell type-dependent manner. Anat. Sci. Int..

[16] Beach T.G., Adler C.H., Sue L.I., Vedders L., Lue L., White Iii C.L., Akiyama H., Caviness J.N., Shill H.A., Sabbagh M.N. (2010). Multi-organ distribution of phosphorylated alpha-synuclein histopathology in subjects with Lewy body disorders. Acta Neuropathol..

[17] Fumimura Y., Ikemura M., Saito Y., Sengoku R., Kanemaru K., Sawabe M., Arai T., Ito G., Iwatsubo T., Fukayama M. (2007). Analysis of the adrenal gland is useful for evaluating pathology of the peripheral autonomic nervous system in lewy body disease. J. Neuropathol. Exp. Neurol..

[18] Wakabayashi K., Mori F., Tanji K., Orimo S., Takahashi H. (2010). Involvement of the peripheral nervous system in synucleinopathies, tauopathies and other neurodegenerative proteinopathies of the brain. Acta Neuropathol..

[19] Del Tredici K., Hawkes C.H., Ghebremedhin E., Braak H. (2010). Lewy pathology in the submandibular gland of individuals with incidental Lewy body disease and sporadic Parkinson’s disease. Acta Neuropathol..

[20] Cersosimo M.G. (2015). Gastrointestinal biopsies for the diagnosis of alpha-synuclein pathology in Parkinson’s disease. Gastroenterol. Res. Pract..

[21] Emamzadeh F.N. (2016). Alpha-synuclein structure, functions, and interactions. J. Res. Med. Sci..

[22] Dehay B., Bourdenx M., Gorry P., Przedborski S., Vila M., Hunot S., Singleton A., Olanow C.W., Merchant K.M., Bezard E. (2015). Targeting α-synuclein for treatment of Parkinson’s disease: Mechanistic and therapeutic considerations. Lancet Neurol..

[23] Waxman E.A., Mazzulli J.R., Giasson B.I. (2009). Characterization of hydrophobic residue requirements for alpha-synuclein fibrillization. Biochemistry.

[24] Mehra S., Sahay S., Maji S.K. (2019). α-Synuclein misfolding and aggregation: Implications in Parkinson’s disease pathogenesis. Biochim. Biophys. Acta Proteins Proteom..

[25] Allen Reish H.E., Standaert D.G. (2015). Role of α-synuclein in inducing innate and adaptive immunity in Parkinson disease. J. Parkinsons’ Dis..

[26] Kahle P.J., Neumann M., Ozmen L., Muller V., Jacobsen H., Schindzielorz A., Okochi M., Leimer U., van Der Putten H., Probst A. (2000). Subcellular localization of wild-type and Parkinson’s disease-associated mutant alpha -synuclein in human and transgenic mouse brain. J. Neurosci..

[27] Davidson W.S., Jonas A., Clayton D.F., George J.M. (1998). Stabilization of alpha-synuclein secondary structure upon binding to synthetic membranes. J. Biol. Chem..

[28] Varkey J., Isas J.M., Mizuno N., Jensen M.B., Bhatia V.K., Jao C.C., Petrlova J., Voss J.C., Stamou D.G., Steven A.C. (2010). Membrane curvature induction and tubulation are common features of synucleins and apolipoproteins. J. Biol. Chem..

[29] Burré J., Sharma M., Tsetsenis T., Buchman V., Etherton M.R., Südhof T.C. (2010). Alpha-synuclein promotes SNARE-complex assembly in vivo and in vitro. Science.

[30] Gould N., Mor D.E., Lightfoot R., Malkus K., Giasson B., Ischiropoulos H. (2014). Evidence of native α-synuclein conformers in the human brain. J. Biol. Chem..

[31] Bartels T., Choi J.G., Selkoe D.J. (2011). α-Synuclein occurs physiologically as a helically folded tetramer that resists aggregation. Nature.

[32] Dettmer U., Newman A.J., Soldner F., Luth E.S., Kim N.C., von Saucken V.E., Sanderson J.B., Jaenisch R., Bartels T., Selkoe D. (2015). Parkinson-causing α-synuclein missense mutations shift native tetramers to monomers as a mechanism for disease initiation. Nat. Commun..

[33] Polymeropoulos M.H., Lavedan C., Leroy E., Ide S.E., Dehejia A., Dutra A., Pike B., Root H., Rubenstein J., Boyer R. (1997). Mutation in the alpha-synuclein gene identified in families with Parkinson’s disease. Science.

[34] Krüger R., Kuhn W., Müller T., Woitalla D., Graeber M., Kösel S., Przuntek H., Epplen J.T., Schöls L., Riess O. (1998). Ala30Pro mutation in the gene encoding alpha-synuclein in Parkinson’s disease. Nat. Genet..

[35] Zarranz J.J., Alegre J., Gómez-Esteban J.C., Lezcano E., Ros R., Ampuero I., Vidal L., Hoenicka J., Rodriguez O., Atarés B. (2004). The new mutation, E46K, of alpha-synuclein causes Parkinson and Lewy body dementia. Ann. Neurol..

[36] Appel-Cresswell S., Vilarino-Guell C., Encarnacion M., Sherman H., Yu I., Shah B., Weir D., Thompson C., Szu-Tu C., Trinh J. (2013). Alpha-synuclein p.H50Q, a novel pathogenic mutation for Parkinson’s disease. Mov. Disord..

[37] Proukakis C., Dudzik C.G., Brier T., MacKay D.S., Cooper J.M., Millhauser G.L., Houlden H., Schapira A.H. (2013). A novel α-synuclein missense mutation in Parkinson disease. Neurology.

[38] Lesage S., Anheim M., Letournel F., Bousset L., Honoré A., Rozas N., Pieri L., Madiona K., Dürr A., Melki R. (2013). G51D α-synuclein mutation causes a novel parkinsonian-pyramidal syndrome. Ann. Neurol..

[39] Pasanen P., Myllykangas L., Siitonen M., Raunio A., Kaakkola S., Lyytinen J., Tienari P.J., Pöyhönen M., Paetau A. (2014). Novel α-synuclein mutation A53E associated with atypical multiple system atrophy and Parkinson’s disease-type pathology. Neurobiol. Aging.

[40] Singleton A.B., Farrer M., Johnson J., Singleton A., Hague S., Kachergus J., Hulihan M., Peuralinna T., Dutra A., Nussbaum R. (2003). Alpha-synuclein locus triplication causes Parkinson’s disease. Science.

[41] Chartier-Harlin M.-C., Kachergus J., Roumier C., Mouroux V., Douay X., Lincoln S., Levecque C., Larvor L., Andrieux J., Hulihan M. (2004). Alpha-synuclein locus duplication as a cause of familial Parkinson’s disease. Lancet.

[42] Iwatsubo T. (2007). Pathological biochemistry of alpha-synucleinopathy. Neuropathology.

[43] Gallegos S., Pacheco C., Peters C., Opazo C.M., Aguayo L.G. (2015). Features of alpha-synuclein that could explain the progression and irreversibility of Parkinson’s disease. Front. Neurosci..

[44] Braak H., Del Tredici K., Rüb U., de Vos R.A., Jansen Steur E.N., Braak E. (2003). Staging of brain pathology related to sporadic Parkinson’s disease. Neurobiol. Aging.

[45] Li J.Y., Englund E., Holton J.L., Soulet D., Hagell P., Lees A.J., Lashley T., Quinn N.P., Rehncrona S., Bjorklund A. (2008). Lewy bodies in grafted neurons in subjects with Parkinson’s disease suggest host-to-graft disease propagation. Nat. Med..

[46] Kordower J.H., Chu Y., Hauser R.A., Freeman T.B., Olanow C.W. (2008). Lewy body-like pathology in long-term embryonic nigral transplants in Parkinson’s disease. Nat. Med..

[47] Lee H.J., Patel S., Lee S.J. (2005). Intravesicular localization and exocytosis of alpha-synuclein and its aggregates. J. Neurosci..

[48] Danzer K.M., Kranich L.R., Ruf W.P., Cagsal-Getkin O., Winslow A.R., Zhu L., Vanderburg C.R., McLean P.J. (2012). Exosomal cell-to-cell transmission of alpha synuclein oligomers. Mol. Neurodegener..

[49] Alvarez-Erviti L., Seow Y., Schapira A.H., Gardiner C., Sargent I.L., Wood M.J., Cooper J.M. (2011). Lysosomal dysfunction increases exosome-mediated alpha-synuclein release and transmission. Neurobiol. Dis..

[50] Emmanouilidou E., Melachroinou K., Roumeliotis T., Garbis S.D., Ntzouni M., Margaritis L.H., Stefanis L., Vekrellis K. (2010). Cell-produced alpha-synuclein is secreted in a calcium-dependent manner by exosomes and impacts neuronal survival. J. Neurosci..

[51] Tsigelny I.F., Sharikov Y., Wrasidlo W., Gonzalez T., Desplats P.A., Crews L., Spencer B., Masliah E. (2012). Role of α-synuclein penetration into the membrane in the mechanisms of oligomer pore formation. FEBS J..

[52] Jang A., Lee H.J., Suk J.E., Jung J.W., Kim K.P., Lee S.J. (2010). Non-classical exocytosis of alpha-synuclein is sensitive to folding states and promoted under stress conditions. J. Neurochem..

[53] Rodriguez L., Marano M.M., Tandon A. (2018). Import and export of misfolded α-synuclein. Front. Neurosci..

[54] Abounit S., Bousset L., Loria F., Zhu S., de Chaumont F., Pieri L., Olivo-Marin J.C., Melki R., Zurzolo C. (2016). Tunneling nanotubes spread fibrillar α-synuclein by intercellular trafficking of lysosomes. EMBO J..

[55] Flavin W.P., Bousset L., Green Z.C., Chu Y., Skarpathiotis S., Chaney M.J., Kordower J.H., Melki R., Campbell E.M. (2017). Endocytic vesicle rupture is a conserved mechanism of cellular invasion by amyloid proteins. Acta Neuropathol..

[56] Holmes B.B., DeVos S.L., Kfoury N., Li M., Jacks R., Yanamandra K., Ouidja M.O., Brodsky F.M., Marasa J., Bagchi D.P. (2013). Heparan sulfate proteoglycans mediate internalization and propagation of specific proteopathic seeds. Proc. Natl. Acad. Sci. USA.

[57] Mao X., Ou M.T., Karuppagounder S.S., Kam T.I., Yin X., Xiong Y., Ge P., Umanah G.E., Brahmachari S., Shin J.H. (2016). Pathological α-synuclein transmission initiated by binding lymphocyte-activation gene 3. Science.

[58] Luk K.C., Song C., O’Brien P., Stieber A., Branch J.R., Brunden K.R., Trojanowski J.Q., Lee V.M. (2009). Exogenous alpha-synuclein fibrils seed the formation of Lewy body-like intracellular inclusions in cultured cells. Proc. Natl. Acad. Sci. USA.

[59] Volpicelli-Daley L.A., Luk K.C., Patel T.P., Tanik S.A., Riddle D.M., Stieber A., Meaney D.F., Trojanowski J.Q., Lee V.M. (2011). Exogenous α-synuclein fibrils induce Lewy body pathology leading to synaptic dysfunction and neuron death. Neuron.

[60] Wong Y.C., Krainc D. (2017). α-synuclein toxicity in neurodegeneration: Mechanism and therapeutic strategies. Nat. Med..

[61] Choi B.K., Choi M.G., Kim J.Y., Yang Y., Lai Y., Kweon D.H., Lee N.K., Shin Y.K. (2013). Large α-synuclein oligomers inhibit neuronal SNARE-mediated vesicle docking. Proc. Natl. Acad. Sci. USA.

[62] Nemani V.M., Lu W., Berge V., Nakamura K., Onoa B., Lee M.K., Chaudhry F.A., Nicoll R.A., Edwards R.H. (2010). Increased expression of alpha-synuclein reduces neurotransmitter release by inhibiting synaptic vesicle reclustering after endocytosis. Neuron.

[63] Maroteaux L., Campanelli J.T., Scheller R.H. (1988). Synuclein: A neuron-specific protein localized to the nucleus and presynaptic nerve terminal. J. Neurosci..

[64] Xu S., Zhou M., Yu S., Cai Y., Zhang A., Uéda K., Chan P. (2006). Oxidative stress induces nuclear translocation of C-terminus of alpha-synuclein in dopaminergic cells. Biochem. Biophys. Res. Commun..

[65] Schell H., Hasegawa T., Neumann M., Kahle P.J. (2009). Nuclear and neuritic distribution of serine-129 phosphorylated alpha-synuclein in transgenic mice. Neuroscience.

[66] Kontopoulos E., Parvin J.D., Feany M.B. (2006). Alpha-synuclein acts in the nucleus to inhibit histone acetylation and promote neurotoxicity. Hum. Mol. Genet..

[67] Rousseaux M.W., de Haro M., Lasagna-Reeves C.A., De Maio A., Park J., Jafar-Nejad P., Al-Ramahi I., Sharma A., See L., Lu N. (2016). TRIM28 regulates the nuclear accumulation and toxicity of both alpha-synuclein and tau. eLife.

[68] Siddiqui A., Chinta S.J., Mallajosyula J.K., Rajagopolan S., Hanson I., Rane A., Melov S., Andersen J.K. (2012). Selective binding of nuclear alpha-synuclein to the PGC1alpha promoter under conditions of oxidative stress may contribute to losses in mitochondrial function: Implications for Parkinson’s disease. Free Radic. Biol. Med..

[69] Eschbach J., von Einem B., Müller K., Bayer H., Scheffold A., Morrison B.E., Rudolph K.L., Thal D.R., Witting A., Weydt P. (2015). Mutual exacerbation of peroxisome proliferator-activated receptor γ coactivator 1α deregulation and α-synuclein oligomerization. Ann. Neurol..

[70] Luth E.S., Stavrovskaya I.G., Bartels T., Kristal B.S., Selkoe D.J. (2014). Soluble, prefibrillar α-synuclein oligomers promote complex I-dependent, Ca2+-induced mitochondrial dysfunction. J. Biol. Chem..

[71] Zaichick S.V., McGrath K.M., Caraveo G. (2017). The role of Ca^2+^ signaling in Parkinson’s disease. Dis. Models Mech..

[72] Betzer C., Lassen L.B., Olsen A., Kofoed R.H., Reimer L., Gregersen E., Zheng J., Cali T., Gai W.P., Chen T. (2018). Alpha-synuclein aggregates activate calcium pump SERCA leading to calcium dysregulation. EMBO Rep..

[73] Caraveo G., Auluck P.K., Whitesell L., Chung C.Y., Baru V., Mosharov E.V., Yan X., Ben-Johny M., Soste M., Picotti P. (2014). Calcineurin determines toxic versus beneficial responses to α-synuclein. Proc. Natl. Acad. Sci. USA.

[74] Luo J., Sun L., Lin X., Liu G., Yu J., Parisiadou L., Xie C., Ding J., Cai H. (2014). A calcineurin- and NFAT-dependent pathway is involved in α-synuclein-induced degeneration of midbrain dopaminergic neurons. Hum. Mol. Genet..

[75] Volles M.J., Lansbury P.T. (2002). Vesicle permeabilization by protofibrillar alpha-synuclein is sensitive to Parkinson’s disease-linked mutations and occurs by a pore-like mechanism. Biochemistry.

[76] Van Rooijen B.D., Claessens M.M., Subramaniam V. (2010). Membrane permeabilization by oligomeric α-synuclein: In search of the mechanism. PLoS ONE.

[77] Mazzulli J.R., Xu Y.H., Sun Y., Knight A.L., McLean P.J., Caldwell G.A., Sidransky E., Grabowski G.A., Krainc D. (2011). Gaucher disease glucocerebrosidase and α-synuclein form a bidirectional pathogenic loop in synucleinopathies. Cell.

[78] Mazzulli J.R., Zunke F., Isacson O., Studer L., Krainc D. (2016). α-Synuclein-induced lysosomal dysfunction occurs through disruptions in protein trafficking in human midbrain synucleinopathy models. Proc. Natl. Acad. Sci. USA.

[79] Wong Y.C., Krainc D. (2016). Lysosomal trafficking defects link Parkinson’s disease with Gaucher’s disease. Mov. Disord..

[80] (2018). The UniProt Consortium. UniProt: The universal protein knowledgebase. Nucleic Acids Res..

[81] Kitada T., Asakawa S., Hattori N., Matsumine H., Yamamura Y., Minoshima S., Yokochi M., Mizuno Y., Shimizu N. (1998). Mutations in the parkin gene cause autosomal recessive juvenile parkinsonism. Nature.

[82] Hedrich K., Eskelson C., Wilmot B., Marder K., Harris J., Garrels J., Meija-Santana H., Vieregge P., Jacobs H., Bressman S.B. (2004). Distribution, type, and origin of Parkin mutations: Review and case studies. Mov. Disord..

[83] Giguère N., Pacelli C., Saumure C., Bourque M.-J., Matheoud D., Levesque D., Slack R.S., Park D.S., Trudeau L.-É. (2018). Comparative analysis of Parkinson’s disease-associated genes in mice reveals altered survival and bioenergetics of Parkin-deficient dopamine neurons. J. Biol. Chem..

[84] Geisler S., Holmstrom K.M., Treis A., Skujat D., Weber S.S., Fiesel F.C., Kahle P.J., Springer W. (2010). The PINK1/Parkin-mediated mitophagy is compromised by PD-associated mutations. Autophagy.

[85] Spratt D.E., Walden H., Shaw G.S. (2014). RBR E3 ubiquitin ligases: New structures, new insights, new questions. Biochem. J..

[86] Sarraf S.A., Raman M., Guarani-Pereira V., Sowa M.E., Huttlin E.L., Gygi S.P., Harper J.W. (2013). Landscape of the PARKIN-dependent ubiquitylome in response to mitochondrial depolarization. Nature.

[87] Hristova V.A., Beasley S.A., Rylett R.J., Shaw G.S. (2009). Identification of a novel Zn2+-binding domain in the autosomal recessive juvenile Parkinson-related E3 ligase parkin. J. Biol. Chem..

[88] Truban D., Hou X., Caulfield T.R., Fiesel F.C., Springer W. (2017). PINK1, Parkin, and mitochondrial quality control: What can we learn about Parkinson’s disease pathobiology?. J. Parkinson’s Dis..

[89] Lesage S., Magali P., Lohmann E., Lacomblez L., Teive H., Janin S., Cousin P.-Y., Dürr A., Brice A., French Parkinson Disease Genetics Study G. (2007). Deletion of the parkin and PACRG gene promoter in early-onset parkinsonism. Hum. Mutat..

[90] Chung K.K.K., Thomas B., Li X., Pletnikova O., Troncoso J.C., Marsh L., Dawson V.L., Dawson T.M. (2004). S-nitrosylation of parkin regulates ubiquitination and compromises parkin’s protective function. Science.

[91] Imam S.Z., Zhou Q., Yamamoto A., Valente A.J., Ali S.F., Bains M., Roberts J.L., Kahle P.J., Clark R.A., Li S. (2011). Novel regulation of parkin function through c-Abl-mediated tyrosine phosphorylation: Implications for Parkinson’s disease. J. Neurosci..

[92] Ko H.S., Lee Y., Shin J.-H., Karuppagounder S.S., Gadad B.S., Koleske A.J., Pletnikova O., Troncoso J.C., Dawson V.L., Dawson T.M. (2010). Phosphorylation by the c-Abl protein tyrosine kinase inhibits parkin’s ubiquitination and protective function. Proc. Natl. Acad. Sci. USA.

[93] LaVoie M.J., Ostaszewski B.L., Weihofen A., Schlossmacher M.G., Selkoe D.J. (2005). Dopamine covalently modifies and functionally inactivates parkin. Nat. Med..

[94] Meng F., Yao D., Shi Y., Kabakoff J., Wu W., Reicher J., Ma Y., Moosmann B., Masliah E., Lipton S.A. (2011). Oxidation of the cysteine-rich regions of parkin perturbs its E3 ligase activity and contributes to protein aggregation. Mol. Neurodegener..

[95] Wang C., Ko H.S., Thomas B., Tsang F., Chew K.C.M., Tay S.-P., Ho M.W.L., Lim T.-M., Soong T.-W., Pletnikova O. (2005). Stress-induced alterations in parkin solubility promote parkin aggregation and compromise parkin’s protective function. Hum. Mol. Genet..

[96] Yao D., Gu Z., Nakamura T., Shi Z.-Q., Ma Y., Gaston B., Palmer L.A., Rockenstein E.M., Zhang Z., Masliah E. (2004). Nitrosative stress linked to sporadic Parkinson’s disease: S-nitrosylation of parkin regulates its E3 ubiquitin ligase activity. Proc. Natl. Acad. Sci. USA.

[97] Corti O., Lesage S., Brice A. (2011). What genetics tells us about the causes and mechanisms of Parkinson’s disease. Physiol. Rev..

[98] Lücking C.B., Dürr A., Bonifati V., Vaughan J., De Michele G., Gasser T., Harhangi B.S., Meco G., Denèfle P., Wood N.W. (2000). Association between early-onset Parkinson’s disease and mutations in the parkin gene. N. Engl. J. Med..

[99] Schulte C., Gasser T. (2011). Genetic basis of Parkinson’s disease: Inheritance, penetrance, and expression. Appl. Clin. Genet..

[100] Lohmann E., Thobois S., Lesage S., Broussolle E., du Montcel S.T., Ribeiro M.J., Remy P., Pelissolo A., Dubois B., Mallet L. (2009). A multidisciplinary study of patients with early-onset PD with and without parkin mutations. Neurology.

[101] Mori H., Kondo T., Yokochi M., Matsumine H., Nakagawa-Hattori Y., Miyake T., Suda K., Mizuno Y. (1998). Pathologic and biochemical studies of juvenile parkinsonism linked to chromosome 6q. Neurology.

[102] Wasner K., Grünewald A., Klein C. (2020). Parkin-linked Parkinson’s disease: From clinical insights to pathogenic mechanisms and novel therapeutic approaches. Neurosci. Res..

[103] Khan N.L., Graham E., Critchley P., Schrag A.E., Wood N.W., Lees A.J., Bhatia K.P., Quinn N. (2003). Parkin disease: A phenotypic study of a large case series. Brain J. Neurol..

[104] Hampe C., Ardila-Osorio H., Fournier M., Brice A., Corti O. (2006). Biochemical analysis of Parkinson’s disease-causing variants of Parkin, an E3 ubiquitin-protein ligase with monoubiquitylation capacity. Hum. Mol. Genet..

[105] Matsuda N., Kitami T., Suzuki T., Mizuno Y., Hattori N., Tanaka K. (2006). Diverse effects of pathogenic mutations of Parkin that catalyze multiple monoubiquitylation in vitro. J. Biol. Chem..

[106] Doss-Pepe E.W., Chen L., Madura K. (2005). Alpha-synuclein and parkin contribute to the assembly of ubiquitin lysine 63-linked multiubiquitin chains. J. Biol. Chem..

[107] Chan N.C., Salazar A.M., Pham A.H., Sweredoski M.J., Kolawa N.J., Graham R.L.J., Hess S., Chan D.C. (2011). Broad activation of the ubiquitin-proteasome system by Parkin is critical for mitophagy. Hum. Mol. Genet..

[108] Zheng X., Hunter T. (2013). Parkin mitochondrial translocation is achieved through a novel catalytic activity coupled mechanism. Cell Res..

[109] Seirafi M., Kozlov G., Gehring K. (2015). Parkin structure and function. FEBS J..

[110] Lim K.L., Tan J.M. (2007). Role of the ubiquitin proteasome system in Parkinson’s disease. BMC Biochem..

[111] Bard J.A.M., Goodall E.A., Greene E.R., Jonsson E., Dong K.C., Martin A. (2018). Structure and function of the 26S Proteasome. Annu. Rev. Biochem..

[112] Geisler S., Holmström K.M., Skujat D., Fiesel F.C., Rothfuss O.C., Kahle P.J., Springer W. (2010). PINK1/Parkin-mediated mitophagy is dependent on VDAC1 and p62/SQSTM1. Nat. Cell Biol..

[113] Narendra D., Tanaka A., Suen D.-F., Youle R.J. (2008). Parkin is recruited selectively to impaired mitochondria and promotes their autophagy. J. Cell Biol..

[114] Narendra D., Tanaka A., Suen D.-F., Youle R.J. (2009). Parkin-induced mitophagy in the pathogenesis of Parkinson disease. Autophagy.

[115] Matsuda N., Sato S., Shiba K., Okatsu K., Saisho K., Gautier C.A., Sou Y.-S., Saiki S., Kawajiri S., Sato F. (2010). PINK1 stabilized by mitochondrial depolarization recruits Parkin to damaged mitochondria and activates latent Parkin for mitophagy. J. Cell Biol..

[116] Harper J.W., Ordureau A., Heo J.-M. (2018). Building and decoding ubiquitin chains for mitophagy. Nat. Rev. Mol. Cell Biol..

[117] Becker D., Richter J., Tocilescu M.A., Przedborski S., Voos W. (2012). Pink1 kinase and its membrane potential (Deltaψ)-dependent cleavage product both localize to outer mitochondrial membrane by unique targeting mode. J. Biol. Chem..

[118] Yamano K., Youle R.J. (2013). PINK1 is degraded through the N-end rule pathway. Autophagy.

[119] Greene A.W., Grenier K., Aguileta M.A., Muise S., Farazifard R., Haque M.E., McBride H.M., Park D.S., Fon E.A. (2012). Mitochondrial processing peptidase regulates PINK1 processing, import and Parkin recruitment. EMBO Rep..

[120] Narendra D.P., Jin S.M., Tanaka A., Suen D.-F., Gautier C.A., Shen J., Cookson M.R., Youle R.J. (2010). PINK1 is selectively stabilized on impaired mitochondria to activate Parkin. PLoS Biol..

[121] Kondapalli C., Kazlauskaite A., Zhang N., Woodroof H.I., Campbell D.G., Gourlay R., Burchell L., Walden H., Macartney T.J., Deak M. (2012). PINK1 is activated by mitochondrial membrane potential depolarization and stimulates Parkin E3 ligase activity by phosphorylating Serine 65. Open Biol..

[122] Shiba-Fukushima K., Imai Y., Yoshida S., Ishihama Y., Kanao T., Sato S., Hattori N. (2012). PINK1-mediated phosphorylation of the Parkin ubiquitin-like domain primes mitochondrial translocation of Parkin and regulates mitophagy. Sci. Rep..

[123] Weil R., Laplantine E., Curic S., Génin P. (2018). Role of optineurin in the mitochondrial dysfunction: Potential implications in neurodegenerative diseases and cancer. Front. Immunol..

[124] Park J.S., Davis R.L., Sue C.M. (2018). Mitochondrial dysfunction in Parkinson’s disease: New mechanistic insights and therapeutic perspectives. Curr. Neurol. Neurosci. Rep..

[125] Hang L., Thundyil J., Lim K.-L. (2015). Mitochondrial dysfunction and Parkinson disease: A Parkin-AMPK alliance in neuroprotection. Ann. N. Y. Acad. Sci..

[126] Shin J.-H., Ko H.S., Kang H., Lee Y., Lee Y.-I., Pletinkova O., Troconso J.C., Dawson V.L., Dawson T.M. (2011). PARIS (ZNF746) repression of PGC-1α contributes to neurodegeneration in Parkinson’s disease. Cell.

[127] Stevens D.A., Lee Y., Kang H.C., Lee B.D., Lee Y.I., Bower A., Jiang H., Kang S.U., Andrabi S.A., Dawson V.L. (2015). Parkin loss leads to PARIS-dependent declines in mitochondrial mass and respiration. Proc. Natl. Acad. Sci. USA.

[128] Bogetofte H., Jensen P., Ryding M., Schmidt S.I., Okarmus J., Ritter L., Worm C.S., Hohnholt M.C., Azevedo C., Roybon L. (2019). PARK2 mutation causes metabolic disturbances and impaired survival of human iPSC-derived neurons. Front. Cell. Neurosci..

[129] Klein C., Lohmann-Hedrich K., Rogaeva E., Schlossmacher M.G., Lang A.E. (2007). Deciphering the role of heterozygous mutations in genes associated with parkinsonism. Lancet Neurol..

[130] Huttenlocher J., Stefansson H., Steinberg S., Helgadottir H.T., Sveinbjörnsdóttir S., Riess O., Bauer P., Stefansson K. (2015). Heterozygote carriers for CNVs in PARK2 are at increased risk of Parkinson’s disease. Hum. Mol. Genet..

[131] Farrer M., Chan P., Chen R., Tan L., Lincoln S., Hernandez D., Forno L., Gwinn-Hardy K., Petrucelli L., Hussey J. (2001). Lewy bodies and parkinsonism in families with parkin mutations. Ann. Neurol..

[132] Pramstaller P.P., Schlossmacher M.G., Jacques T.S., Scaravilli F., Eskelson C., Pepivani I., Hedrich K., Adel S., Gonzales-McNeal M., Hilker R. (2005). Lewy body Parkinson’s disease in a large pedigree with 77 Parkin mutation carriers. Ann. Neurol..

[133] Ruffmann C., Zini M., Goldwurm S., Bramerio M., Spinello S., Rusconi D., Gambacorta M., Tagliavini F., Pezzoli G., Giaccone G. (2012). Lewy body pathology and typical Parkinson disease in a patient with a heterozygous (R275W) mutation in the Parkin gene (PARK2). Acta Neuropathol..

[134] Miyakawa S., Ogino M., Funabe S., Uchino A., Shimo Y., Hattori N., Ichinoe M., Mikami T., Saegusa M., Nishiyama K. (2013). Lewy body pathology in a patient with a homozygous parkin deletion. Mov. Disord..

[135] Doherty K.M., Silveira-Moriyama L., Parkkinen L., Healy D.G., Farrell M., Mencacci N.E., Ahmed Z., Brett F.M., Hardy J., Quinn N. (2013). Parkin disease: A clinicopathologic entity?. JAMA Neurol..

[136] Selikhova M., Kempster P.A., Revesz T., Holton J.L., Lees A.J. (2013). Neuropathological findings in benign tremulous parkinsonism. Mov. Disord..

[137] Sharp M.E., Marder K.S., Côté L., Clark L.N., Nichols W.C., Vonsattel J.-P., Alcalay R.N. (2014). Parkinson’s disease with Lewy bodies associated with a heterozygous PARKIN dosage mutation. Mov. Disord..

[138] Sasaki S., Shirata A., Yamane K., Iwata M. (2004). Parkin-positive autosomal recessive juvenile Parkinsonism with alpha-synuclein-positive inclusions. Neurology.

[139] Takahashi H., Ohama E., Suzuki S., Horikawa Y., Ishikawa A., Morita T., Tsuji S., Ikuta F. (1994). Familial juvenile parkinsonism: Clinical and pathologic study in a family. Neurology.

[140] Yamamura Y., Arihiro K., Kohriyama T., Nakamura S. (1993). Early-onset parkinsonism with diurnal fluctuation—Clinical and pathological studies. Rinsho Shinkeigaku.

[141] Yamamura Y., Kuzuhara S., Kondo K., Yanagi T., Uchida M., Matsumine H., Mizuno Y. (1998). Clinical, pathologic and genetic studies on autosomal recessive early-onset parkinsonism with diurnal fluctuation. Parkinsonism Relat. Disord..

[142] Hayashi S., Wakabayashi K., Ishikawa A., Nagai H., Saito M., Maruyama M., Takahashi T., Ozawa T., Tsuji S., Takahashi H. (2000). An autopsy case of autosomal-recessive juvenile parkinsonism with a homozygous exon 4 deletion in the parkin gene. Mov. Disord..

[143] Van de Warrenburg B.P., Lammens M., Lücking C.B., Denèfle P., Wesseling P., Booij J., Praamstra P., Quinn N., Brice A., Horstink M.W. (2001). Clinical and pathologic abnormalities in a family with parkinsonism and parkin gene mutations. Neurology.

[144] Gouider-Khouja N., Larnaout A., Amouri R., Sfar S., Belal S., Ben Hamida C., Ben Hamida M., Hattori N., Mizuno Y., Hentati F. (2003). Autosomal recessive parkinsonism linked to parkin gene in a Tunisian family. Clinical, genetic and pathological study. Parkinsonism Relat. Disord..

[145] Orimo S., Amino T., Yokochi M., Kojo T., Uchihara T., Takahashi A., Wakabayashi K., Takahashi H., Hattori N., Mizuno Y. (2005). Preserved cardiac sympathetic nerve accounts for normal cardiac uptake of MIBG in PARK2. Mov. Disord..

[146] Cornejo-Olivas M.R., Torres L., Mata I.F., Mazzetti P., Rivas D., Cosentino C., Inca-Martinez M., Cuba J.M., Zabetian C.P., Leverenz J.B. (2015). A Peruvian family with a novel PARK2 mutation: Clinical and pathological characteristics. Parkinsonism Relat. Disord..

[147] Johansen K.K., Torp S.H., Farrer M.J., Gustavsson E.K., Aasly J.O. (2018). A case of Parkinson’s disease with no Lewy body pathology due to a homozygous exon deletion in Parkin. Case Rep. Neurol. Med..

[148] Yamamura Y. (2010). The long journey to the discovery of PARK2: The 50th anniversary of Japanese society of neuropathology. Neuropathology.

[149] Yamamura Y., Sobue I., Ando K., Iida M., Yanagi T. (1973). Paralysis agitans of early onset with marked diurnal fluctuation of symptoms. Neurology.

[150] Matsumine H., Saito M., Shimoda-Matsubayashi S., Tanaka H., Ishikawa A., Nakagawa-Hattori Y., Yokochi M., Kobayashi T., Igarashi S., Takano H. (1997). Localization of a gene for an autosomal recessive form of juvenile Parkinsonism to chromosome 6q25.2-27. Am. J. Hum. Genet..

[151] Hattori N., Mizuno Y. (2017). Twenty years since the discovery of the parkin gene. J. Neural Transm. (Vienna Austria 1996).

[152] Dickson D.W., Braak H., Duda J.E., Duyckaerts C., Gasser T., Halliday G.M., Hardy J., Leverenz J.B., Del Tredici K., Wszolek Z.K. (2009). Neuropathological assessment of Parkinson’s disease: Refining the diagnostic criteria. Lancet Neurol..

[153] Daniel S.E., Lees A.J. (1993). Parkinson’s disease society brain bank, London: Overview and research. J. Neural Transm. Suppl..

[154] Gelb D.J., Oliver E., Gilman S. (1999). Diagnostic criteria for Parkinson disease. Arch. Neurol..

[155] Adler C.H., Beach T.G., Hentz J.G., Shill H.A., Caviness J.N., Driver-Dunckley E., Sabbagh M.N., Sue L.I., Jacobson S.A., Belden C.M. (2014). Low clinical diagnostic accuracy of early vs. advanced Parkinson disease: Clinicopathologic study. Neurology.

[156] Schneider S.A., Alcalay R.N. (2017). Neuropathology of genetic synucleinopathies with parkinsonism: Review of the literature. Mov. Disord..

[157] Markesbery W.R., Jicha G.A., Liu H., Schmitt F.A. (2009). Lewy body pathology in normal elderly subjects. J. Neuropathol. Exp. Neurol..

[158] Parkkinen L., Soininen H., Laakso M., Alafuzoff I. (2001). Alpha-synuclein pathology is highly dependent on the case selection. Neuropathol. Appl. Neurobiol..

[159] Fearnley J.M., Lees A.J. (1991). Ageing and Parkinson’s disease: Substantia nigra regional selectivity. Brain J. Neurol..

[160] Doherty K.M., Hardy J. (2013). Parkin disease and the Lewy body conundrum. Mov. Disord..

[161] Beasley S.A., Hristova V.A., Shaw G.S. (2007). Structure of the Parkin in-between-ring domain provides insights for E3-ligase dysfunction in autosomal recessive Parkinson’s disease. Proc. Natl. Acad. Sci. USA.

[162] Sriram S.R., Li X., Ko H.S., Chung K.K., Wong E., Lim K.L., Dawson V.L., Dawson T.M. (2005). Familial-associated mutations differentially disrupt the solubility, localization, binding and ubiquitination properties of parkin. Hum. Mol. Genet..

[163] Cookson M.R., Lockhart P.J., McLendon C., O’Farrell C., Schlossmacher M., Farrer M.J. (2003). RING finger 1 mutations in Parkin produce altered localization of the protein. Hum. Mol. Genet..

[164] Calne D.B., Mizuno Y. (2004). The neuromythology of Parkinson’s disease. Parkinsonism Relat. Disord..

[165] Lim K.L., Chew K.C., Tan J.M., Wang C., Chung K.K., Zhang Y., Tanaka Y., Smith W., Engelender S., Ross C.A. (2005). Parkin mediates nonclassical, proteasomal-independent ubiquitination of synphilin-1: Implications for Lewy body formation. J. Neurosci..

[166] Lim K.L., Dawson V.L., Dawson T.M. (2006). Parkin-mediated lysine 63-linked polyubiquitination: A link to protein inclusions formation in Parkinson’s and other conformational diseases?. Neurobiol. Aging.

[167] Chung K.K., Zhang Y., Lim K.L., Tanaka Y., Huang H., Gao J., Ross C.A., Dawson V.L., Dawson T.M. (2001). Parkin ubiquitinates the alpha-synuclein-interacting protein, synphilin-1: Implications for Lewy-body formation in Parkinson disease. Nat. Med..

[168] Ciechanover A. (2001). Linking ubiquitin, parkin and synphilin-1. Nat. Med..

[169] Lashuel H.A., Overk C.R., Oueslati A., Masliah E. (2013). The many faces of alpha-synuclein: From structure and toxicity to therapeutic target. Nat. Rev. Neurosci..

[170] Anderson J.P., Walker D.E., Goldstein J.M., de Laat R., Banducci K., Caccavello R.J., Barbour R., Huang J., Kling K., Lee M. (2006). Phosphorylation of Ser-129 is the dominant pathological modification of alpha-synuclein in familial and sporadic Lewy body disease. J. Biol. Chem..

[171] Fujiwara H., Hasegawa M., Dohmae N., Kawashima A., Masliah E., Goldberg M.S., Shen J., Takio K., Iwatsubo T. (2002). alpha-synuclein is phosphorylated in synucleinopathy lesions. Nat. Cell Biol..

[172] Nonaka T., Watanabe S.T., Iwatsubo T., Hasegawa M. (2010). Seeded aggregation and toxicity of α-synuclein and tau: Cellular models of neurodegenerative diseases. J. Biol. Chem..

[173] Desplats P., Lee H.J., Bae E.J., Patrick C., Rockenstein E., Crews L., Spencer B., Masliah E., Lee S.J. (2009). Inclusion formation and neuronal cell death through neuron-to-neuron transmission of alpha-synuclein. Proc. Natl. Acad. Sci. USA.

[174] Hansen C., Angot E., Bergström A.L., Steiner J.A., Pieri L., Paul G., Outeiro T.F., Melki R., Kallunki P., Fog K. (2011). α-Synuclein propagates from mouse brain to grafted dopaminergic neurons and seeds aggregation in cultured human cells. J. Clin. Investig..

[175] Luk K.C., Kehm V., Carroll J., Zhang B., O’Brien P., Trojanowski J.Q., Lee V.M. (2012). Pathological α-synuclein transmission initiates Parkinson-like neurodegeneration in nontransgenic mice. Science.

[176] Espay A.J., Vizcarra J.A., Marsili L., Lang A.E., Simon D.K., Merola A., Josephs K.A., Fasano A., Morgante F., Savica R. (2019). Revisiting protein aggregation as pathogenic in sporadic Parkinson and Alzheimer diseases. Neurology.

[177] Zhang J., Li X., Li J.D. (2019). The roles of post-translational modifications on α-synuclein in the pathogenesis of Parkinson’s diseases. Front. Neurosci..

[178] Lee K.W., Chen W., Junn E., Im J.Y., Grosso H., Sonsalla P.K., Feng X., Ray N., Fernandez J.R., Chao Y. (2011). Enhanced phosphatase activity attenuates alpha-synucleinopathy in a mouse model. J. Neurosci..

[179] Park H.J., Lee K.W., Park E.S., Oh S., Yan R., Zhang J., Beach T.G., Adler C.H., Voronkov M., Braithwaite S.P. (2016). Dysregulation of protein phosphatase 2A in parkinson disease and dementia with lewy bodies. Ann. Clin. Transl. Neurol..

[180] Ogris E., Du X., Nelson K.C., Mak E.K., Yu X.X., Lane W.S., Pallas D.C. (1999). A protein phosphatase methylesterase (PME-1) is one of several novel proteins stably associating with two inactive mutants of protein phosphatase 2A. J. Biol. Chem..

[181] Tolstykh T., Lee J., Vafai S., Stock J.B. (2000). Carboxyl methylation regulates phosphoprotein phosphatase 2A by controlling the association of regulatory B subunits. EMBO J..

[182] Khandelwal P.J., Dumanis S.B., Feng L.R., Maguire-Zeiss K., Rebeck G., Lashuel H.A., Moussa C.E. (2010). Parkinson-related parkin reduces alpha-synuclein phosphorylation in a gene transfer model. Mol. Neurodegener..

[183] Burai R., Ait-Bouziad N., Chiki A., Lashuel H.A. (2015). Elucidating the role of site-specific nitration of α-synuclein in the pathogenesis of Parkinson’s disease via protein semisynthesis and mutagenesis. J. Am. Chem. Soc..

[184] Stone D.K., Kiyota T., Mosley R.L., Gendelman H.E. (2012). A model of nitric oxide induced α-synuclein misfolding in Parkinson’s disease. Neurosci. Lett..

[185] Giasson B.I., Duda J.E., Murray I.V., Chen Q., Souza J.M., Hurtig H.I., Ischiropoulos H., Trojanowski J.Q., Lee V.M. (2000). Oxidative damage linked to neurodegeneration by selective alpha-synuclein nitration in synucleinopathy lesions. Science.

[186] Danielson S.R., Held J.M., Schilling B., Oo M., Gibson B.W., Andersen J.K. (2009). Preferentially increased nitration of alpha-synuclein at tyrosine-39 in a cellular oxidative model of Parkinson’s disease. Anal. Chem..

[187] Jiang H., Jiang Q., Liu W., Feng J. (2006). Parkin suppresses the expression of monoamine oxidases. J. Biol. Chem..

[188] Jiang H., Ren Y., Yuen E.Y., Zhong P., Ghaedi M., Hu Z., Azabdaftari G., Nakaso K., Yan Z., Feng J. (2012). Parkin controls dopamine utilization in human midbrain dopaminergic neurons derived from induced pluripotent stem cells. Nat. Commun..

[189] Lee V.M., Goedert M., Trojanowski J.Q. (2001). Neurodegenerative tauopathies. Annu. Rev. Neurosci..

[190] Moussaud S., Jones D.R., Moussaud-Lamodière E.L., Delenclos M., Ross O.A., McLean P.J. (2014). Alpha-synuclein and tau: Teammates in neurodegeneration?. Mol. Neurodegener..

[191] Arima K., Mizutani T., Alim M.A., Tonozuka-Uehara H., Izumiyama Y., Hirai S., Uéda K. (2000). NACP/alpha-synuclein and tau constitute two distinctive subsets of filaments in the same neuronal inclusions in brains from a family of parkinsonism and dementia with Lewy bodies: Double-immunolabeling fluorescence and electron microscopic studies. Acta Neuropathol..

[192] Ishizawa T., Mattila P., Davies P., Wang D., Dickson D.W. (2003). Colocalization of tau and alpha-synuclein epitopes in Lewy bodies. J. Neuropathol. Exp. Neurol..

[193] Biernat J., Gustke N., Drewes G., Mandelkow E.M., Mandelkow E. (1993). Phosphorylation of Ser262 strongly reduces binding of tau to microtubules: Distinction between PHF-like immunoreactivity and microtubule binding. Neuron.

[194] Qureshi H.Y., Paudel H.K. (2011). Parkinsonian neurotoxin 1-methyl-4-phenyl-1,2,3,6-tetrahydropyridine (MPTP) and alpha-synuclein mutations promote Tau protein phosphorylation at Ser262 and destabilize microtubule cytoskeleton in vitro. J. Biol. Chem..

[195] Duka T., Rusnak M., Drolet R.E., Duka V., Wersinger C., Goudreau J.L., Sidhu A. (2006). Alpha-synuclein induces hyperphosphorylation of Tau in the MPTP model of parkinsonism. FASEB J. Off. Publ. Fed. Am. Soc. Exp. Biol..

[196] Meredith G.E., Rademacher D.J. (2011). MPTP mouse models of Parkinson’s disease: An update. J. Parkinsons Dis..

[197] Duka T., Duka V., Joyce J.N., Sidhu A. (2009). Alpha-Synuclein contributes to GSK-3beta-catalyzed Tau phosphorylation in Parkinson’s disease models. FASEB J. Off. Publ. Fed. Am. Soc. Exp. Biol..

[198] Moussa C.E. (2009). Parkin attenuates wild-type tau modification in the presence of beta-amyloid and alpha-synuclein. J. Mol. Neurosci..

[199] Tompkins M.M., Basgall E.J., Zamrini E., Hill W.D. (1997). Apoptotic-like changes in Lewy-body-associated disorders and normal aging in substantia nigral neurons. Am. J. Pathol..

[200] Erekat N.S., Stoker T.B., Greenland J.C. (2018). Apoptosis and its role in Parkinson’s disease. Parkinson’s Disease: Pathogenesis and Clinical Aspects.

[201] Viswanath V., Wu Y., Boonplueang R., Chen S., Stevenson F.F., Yantiri F., Yang L., Beal M.F., Andersen J.K. (2001). Caspase-9 activation results in downstream caspase-8 activation and bid cleavage in 1-methyl-4-phenyl-1,2,3,6-tetrahydropyridine-induced Parkinson’s disease. J. Neurosci..

[202] Mogi M., Togari A., Kondo T., Mizuno Y., Komure O., Kuno S., Ichinose H., Nagatsu T. (2000). Caspase activities and tumor necrosis factor receptor R1 (p55) level are elevated in the substantia nigra from parkinsonian brain. J. Neural Transm. (Vienna Austria 1996).

[203] Hartmann A., Hunot S., Michel P.P., Muriel M.P., Vyas S., Faucheux B.A., Mouatt-Prigent A., Turmel H., Srinivasan A., Ruberg M. (2000). Caspase-3: A vulnerability factor and final effector in apoptotic death of dopaminergic neurons in Parkinson’s disease. Proc. Natl. Acad. Sci. USA.

[204] da Costa C.A., Sunyach C., Giaime E., West A., Corti O., Brice A., Safe S., Abou-Sleiman P.M., Wood N.W., Takahashi H. (2009). Transcriptional repression of p53 by parkin and impairment by mutations associated with autosomal recessive juvenile Parkinson’s disease. Nat. Cell Biol..

[205] Alves da Costa C., Duplan E., Checler F. (2017). α-synuclein and p53 functional interplay in physiopathological contexts. Oncotarget.

[206] Yuan Y., Jin J., Yang B., Zhang W., Hu J., Zhang Y., Chen N.H. (2008). Overexpressed alpha-synuclein regulated the nuclear factor-kappaB signal pathway. Cell. Mol. Neurobiol..

[207] Li D.W., Liu Z.Q., Chen W., Yao M., Li G.R. (2014). Association of glycogen synthase kinase-3β with Parkinson’s disease (review). Mol. Med. Rep..

[208] Linseman D.A., Butts B.D., Precht T.A., Phelps R.A., Le S.S., Laessig T.A., Bouchard R.J., Florez-McClure M.L., Heidenreich K.A. (2004). Glycogen synthase kinase-3beta phosphorylates Bax and promotes its mitochondrial localization during neuronal apoptosis. J. Neurosci..

[209] Watcharasit P., Bijur G.N., Song L., Zhu J., Chen X., Jope R.S. (2003). Glycogen synthase kinase-3beta (GSK3beta) binds to and promotes the actions of p53. J. Biol. Chem..

[210] Maurer U., Charvet C., Wagman A.S., Dejardin E., Green D.R. (2006). Glycogen synthase kinase-3 regulates mitochondrial outer membrane permeabilization and apoptosis by destabilization of MCL-1. Mol. Cell.

[211] Zou H., Li Y., Liu X., Wang X. (1999). An APAF-1.cytochrome c multimeric complex is a functional apoptosome that activates procaspase-9. J. Biol. Chem..

[212] Rosen K.M., Moussa C.E., Lee H.K., Kumar P., Kitada T., Qin G., Fu Q., Querfurth H.W. (2010). Parkin reverses intracellular beta-amyloid accumulation and its negative effects on proteasome function. J. Neurosci. Res..

[213] Hyun D.H., Lee M., Hattori N., Kubo S., Mizuno Y., Halliwell B., Jenner P. (2002). Effect of wild-type or mutant Parkin on oxidative damage, nitric oxide, antioxidant defenses, and the proteasome. J. Biol. Chem..

[214] Ardley H.C., Scott G.B., Rose S.A., Tan N.G., Markham A.F., Robinson P.A. (2003). Inhibition of proteasomal activity causes inclusion formation in neuronal and non-neuronal cells overexpressing Parkin. Mol. Biol. Cell.

[215] Hasegawa M., Fujiwara H., Nonaka T., Wakabayashi K., Takahashi H., Lee V.M., Trojanowski J.Q., Mann D., Iwatsubo T. (2002). Phosphorylated alpha-synuclein is ubiquitinated in alpha-synucleinopathy lesions. J. Biol. Chem..

[216] Videira P.A.Q., Castro-Caldas M. (2018). Linking glycation and glycosylation with inflammation and mitochondrial dysfunction in Parkinson’s disease. Front. Neurosci..

[217] Petrucelli L., O’Farrell C., Lockhart P.J., Baptista M., Kehoe K., Vink L., Choi P., Wolozin B., Farrer M., Hardy J. (2002). Parkin protects against the toxicity associated with mutant alpha-synuclein: Proteasome dysfunction selectively affects catecholaminergic neurons. Neuron.

[218] Yang Y., Nishimura I., Imai Y., Takahashi R., Lu B. (2003). Parkin suppresses dopaminergic neuron-selective neurotoxicity induced by Pael-R in Drosophila. Neuron.

[219] Haywood A.F., Staveley B.E. (2004). Parkin counteracts symptoms in a Drosophila model of Parkinson’s disease. BMC Neurosci..

[220] Lo Bianco C., Schneider B.L., Bauer M., Sajadi A., Brice A., Iwatsubo T., Aebischer P. (2004). Lentiviral vector delivery of parkin prevents dopaminergic degeneration in an alpha-synuclein rat model of Parkinson’s disease. Proc. Natl. Acad. Sci. USA.

[221] Yamada M., Mizuno Y., Mochizuki H. (2005). Parkin gene therapy for alpha-synucleinopathy: A rat model of Parkinson’s disease. Hum. Gene Ther..

[222] Haywood A.F., Staveley B.E. (2006). Mutant alpha-synuclein-induced degeneration is reduced by parkin in a fly model of Parkinson’s disease. Genome.

[223] Yasuda T., Miyachi S., Kitagawa R., Wada K., Nihira T., Ren Y.R., Hirai Y., Ageyama N., Terao K., Shimada T. (2007). Neuronal specificity of alpha-synuclein toxicity and effect of Parkin co-expression in primates. Neuroscience.

[224] Fournier M., Vitte J., Garrigue J., Langui D., Dullin J.P., Saurini F., Hanoun N., Perez-Diaz F., Cornilleau F., Joubert C. (2009). Parkin deficiency delays motor decline and disease manifestation in a mouse model of synucleinopathy. PLoS ONE.

[225] Van Rompuy A.S., Oliveras-Salva M., Van der Perren A., Corti O., Van den Haute C., Baekelandt V. (2015). Nigral overexpression of alpha-synuclein in the absence of parkin enhances alpha-synuclein phosphorylation but does not modulate dopaminergic neurodegeneration. Mol. Neurodegener..

[226] Wilkaniec A., Lenkiewicz A.M., Czapski G.A., Jęśko H.M., Hilgier W., Brodzik R., Gąssowska-Dobrowolska M., Culmsee C., Adamczyk A. (2019). Extracellular alpha-synuclein oligomers induce parkin s-nitrosylation: Relevance to sporadic Parkinson’s disease etiopathology. Mol. Neurobiol..

[227] Goldberg M.S., Fleming S.M., Palacino J.J., Cepeda C., Lam H.A., Bhatnagar A., Meloni E.G., Wu N., Ackerson L.C., Klapstein G.J. (2003). Parkin-deficient mice exhibit nigrostriatal deficits but not loss of dopaminergic neurons. J. Biol. Chem..

[228] Lorenzetti D., Antalffy B., Vogel H., Noveroske J., Armstrong D., Justice M. (2004). The neurological mutant quaking(viable) is Parkin deficient. Mamm. Genome.

[229] Ko H.S., von Coelln R., Sriram S.R., Kim S.W., Chung K.K., Pletnikova O., Troncoso J., Johnson B., Saffary R., Goh E.L. (2005). Accumulation of the authentic parkin substrate aminoacyl-tRNA synthetase cofactor, p38/JTV-1, leads to catecholaminergic cell death. J. Neurosci..

[230] von Coelln R., Thomas B., Andrabi S.A., Lim K.L., Savitt J.M., Saffary R., Stirling W., Bruno K., Hess E.J., Lee M.K. (2006). Inclusion body formation and neurodegeneration are parkin independent in a mouse model of alpha-synucleinopathy. J. Neurosci..

[231] Tanaka K., Suzuki T., Hattori N., Mizuno Y. (2004). Ubiquitin, proteasome and parkin. Biochim. Biophys. Acta.

[232] Narendra D., Walker J.E., Youle R. (2012). Mitochondrial quality control mediated by PINK1 and Parkin: Links to parkinsonism. Cold Spring Harb. Perspect. Biol..

[233] Lim K.L., Ng X.H., Grace L.G., Yao T.P. (2012). Mitochondrial dynamics and Parkinson’s disease: Focus on parkin. Antioxid. Redox Signal..

[234] Palacino J.J., Sagi D., Goldberg M.S., Krauss S., Motz C., Wacker M., Klose J., Shen J. (2004). Mitochondrial dysfunction and oxidative damage in parkin-deficient mice. J. Biol. Chem..

[235] Wakabayashi K., Tanji K., Mori F., Takahashi H. (2007). The Lewy body in Parkinson’s disease: Molecules implicated in the formation and degradation of alpha-synuclein aggregates. Neuropathology.

[236] Terry R.D. (2000). Do neuronal inclusions kill the cell?. J. Neural Transm. Suppl..

[237] Farrer M.J. (2006). Genetics of Parkinson disease: Paradigm shifts and future prospects. Nat. Rev. Genet..

[238] Khandelwal P.J., Moussa C.E. (2010). The Relationship between Parkin and Protein Aggregation in Neurodegenerative Diseases. Front. Psychiatry.

[239] Rodríguez-Navarro J.A., Casarejos M.J., Menéndez J., Solano R.M., Rodal I., Gómez A., Yébenes J.G., Mena M.A. (2007). Mortality, oxidative stress and tau accumulation during ageing in parkin null mice. J. Neurochem..

[240] Hong X., Liu J., Zhu G., Zhuang Y., Suo H., Wang P., Huang D., Xu J., Huang Y., Yu M. (2014). Parkin overexpression ameliorates hippocampal long-term potentiation and β-amyloid load in an Alzheimer’s disease mouse model. Hum. Mol. Genet..

